# SEPT2 crotonylation promotes metastasis and recurrence in hepatocellular carcinoma and is associated with poor survival

**DOI:** 10.1186/s13578-023-00996-7

**Published:** 2023-03-22

**Authors:** Xin-yue Zhang, Ze-xian Liu, Yi-fan Zhang, Li-xia Xu, Meng-ke Chen, Yu-feng Zhou, Jun Yu, Xiao-xing Li, Ning Zhang

**Affiliations:** 1 Department of Gastroenterology and Hepatology, The First Affiliated Hospital, Sun Yat-Sen University, Guangzhou, Guangdong Province China; 2 Institute of Precision Medicine, The First Affiliated Hospital, Sun Yat-Sen University, Guangzhou, Guangdong Province China; 3 State Key Laboratory of Oncology in South China, Collaborative Innovation Center for Cancer Medicine, Sun Yat-Sen University Cancer Center, Guangzhou, China; 4 Department of Liver Surgery, The First Affiliated Hospital, Sun Yat-Sen University, Guangzhou, China; 5 Department of Oncology, The First Affiliated Hospital, Sun Yat-Sen University, Guangzhou, China; 6 Department of Medicine and Therapeutics, Institute of Digestive Disease, State Key Laboratory of Digestive Disease, Li Ka Shing Institute of Health Sciences, CUHK Shenzhen Research Institute, The Chinese University of Hong Kong, Hong Kong SAR, China

**Keywords:** Hepatocellular carcinoma, Posttranslational modification, Crotonylation, SEPT2, Prognosis and recurrence marker

## Abstract

**Background:**

Hepatocellular carcinoma (HCC) metastasis and recurrence lead to therapy failure, which are closely associated with the proteome. However, the role of post-translational modification (PTM) in HCC, especially for the recently discovered lysine crotonylation (Kcr), is elusive.

**Results:**

We investigated the correlation between crotonylation and HCC in 100 tumor tissues and performed stable isotope labeling by amino acids and liquid chromatography tandem mass spectrometry in HCC cells, and we found that crotonylation was positively correlated with HCC metastasis, and higher crotonylation in HCC cells facilitated cell invasiveness. Through bioinformatic analysis, we found that the crotonylated protein SEPT2 was significantly hypercrotonylated in highly invasive cells, while the decrotonylated mutation of SEPT2-K74 impaired SEPT2 GTPase activity and inhibited HCC metastasis in vitro and in vivo. Mechanistically, SIRT2 decrotonylated SEPT2, and P85α was found to be the downstream effector of SEPT2. Moreover, we identified that SEPT2-K74cr was correlated with poor prognosis and recurrence in HCC patients, thus indicating its clinical potential as an independent prognostic factor.

**Conclusions:**

We revealed the role of nonhistone protein crotonylation in regulating HCC metastasis and invasion. Crotonylation facilitated cell invasion through the crotonylated SEPT2-K74-P85α-AKT pathway. High SEPT2-K74 crotonylation predicted poor prognosis and a high recurrence rate in HCC patients. Our study revealed a novel role of crotonylation in promoting HCC metastasis.

**Supplementary Information:**

The online version contains supplementary material available at 10.1186/s13578-023-00996-7.

## Main text

### Introduction

Hepatocellular carcinoma (HCC), comprising 75%-85% of liver cancer cases, is the sixth most commonly diagnosed cancer and the third leading cause of cancer-related death worldwide [1], with less than 20% of 5-year survival rate [2]. Metastasis and recurrence are major obstacles for improving long-term survival of HCC patients. Surgery is currently the most effective treatment of HCC [3], but the recurrence rate remains high with over 70% of 5-year recurrence rate after resection [4]. Therefore, it is important to comprehensively understand the mechanism of HCC metastasis, thereby facilitating the development of novel treatment to improve patient outcomes.

The complex biological functions in humans depend not only on the genome but also the proteome, which is compounded by protein post-translational modifications (PTMs) [5]. Although many studies have elucidated the mechanisms of HCC metastasis and recurrence at genome level, investigations on PTMs, especially for the recently discovered acylation modifications, have been rare. PTM is reversible and could be influenced by various internal and external factors, causing PTM to have more complex and varied features than genome and transcriptome [6]. Changes of proteins properties caused by PTM therefore cannot be fully addressed by simply studying the genome or transcriptome. Given its importance to biological functions, investigations on PTM can provide new insights to cancer research.

Lysine crotonylation (Kcr), which is similar to lysine acetylation (Kac), is a widespread event throughout a cell, and both Kac and Kcr sites overlap on histones [7]. However, Kcr has one more carbon–carbon double bond than Kac. Because of the extended crotonyl hydrocarbon chain, a crotonylated lysine residue is larger and more hydrophobic than an acetylated lysine residue. Moreover, the C–C π-bond in Kcr leads to a rigid and planar protein configuration, which makes it unique among acyl modifications [8]. Functionally, histone Kcr stimulates transcription to a greater extent than Kac [9]. In 2017, Kcr was found in numerous non-histone proteins, suggesting its involvement in multiple cellular functions [10, 11]. Recent studies showed that crotonylation of non-histone proteins ensures accurate spindle positioning in mitosis [12] and is related to aging [13] and colorectal cancer progression [14]. Nevertheless, current understanding of Kcr is far from sufficient, and the in-depth roles of Kcr in biological function and disease progression remain elusive.

In this study, we explored the role of crotonylation in metastasis. Our analysis revealed that crotonylation was positively correlated with EMT in HCC. Septin2 (SEPT2), a member of the septin family of GTPases [15], was hypercrotonylated in highly invasive cells, while de-crotonylation of SEPT2 could impair HCC metastasis. Besides, high level of SEPT2 crotonylation led to poor prognosis and high recurrence rate in HCC patients. Our findings collectively uncover a novel and important feature of crotonylation in promoting HCC metastasis.

## Materials and methods

### Patients and tissues

A total of one hundred formalin-fixed paraffin-embedded tissue blocks from patients diagnosed with HCC undergoing curative surgery at the First Affiliated Hospital of Sun Yat-sen University, Guangzhou, China, in 2014 to 2019, were included in this study. Clinical characteristics were collected from the institutional database. The use of clinical samples was approved by the Institutional Review Board of The First Affiliated Hospital of Sun Yat-sen University (IIT-2022-156). Written informed consent was obtained from each patient. Tissue microarrays (HLivH090Su01, HLivH060CD03) were purchased from Outdo Biotech (Shanghai, China).

### Stable isotope labeling by amino acids in cell culture (SILAC) and liquid chromatography with tandem mass spectrometry (LC–MS/MS)

Cells were labeled with either “heavy isotopic amino acids” (L-13C6-Lysine/L-13C615N4-Arginine) or “light isotopic amino acids” (L-Lysine/L-Arginine) using SILAC Protein Quantitation Kit (Pierce, Thermo) according to manufacturer’s instructions. Cell line was cultured for more than six passages to achieve more than 97% labeling efficiency. Cells were then harvested, and proteins were extracted by trypsin digestion. To enrich crotonylated modified peptides, tryptic peptides were incubated with pre-washed crotonylated antibody beads (PTM-503, PTM Bio, Chicago, IL) and the bound peptides were eluted and vacuum-dried. Tryptic peptides in SILAC were separated by EASY-nLC 1000 UPLC system and subjected to NSI source, followed by tandem mass spectrometry (MS/MS) in Q ExactiveTM Plus (Thermo) coupled online to UPLC.

### Animal experiments

Male NCG mice aged 4 or 5 weeks (19–21 g) were purchased from GemPharmatech Co., Ltd. (Jiangshu, China). Mice were randomly divided into groups and multiple mouse models with different injection approaches of cells were established. For the tail vein injection model, Huh7 cells (3 × 10^6^) in 200 μl PBS were injected into mice tail vein. For the intrasplenic injection model, Huh7 cells (2 × 10^6^) were injected into the spleen, and splenectomy was performed. Mice were sacrificed eight weeks after the injection. For the orthotopic model, Huh7 cells (8 × 10^5^) were injected into the left lobe of liver, and mice were sacrificed 10 weeks later. Tissues were fixed in 10% neutral-buffered formaldehyde for 24 h at room temperature and embedded in paraffin. Fixed tissues were sectioned (5 μm thick), and the standard histological hematoxylin and eosin (H&E) staining was performed. All experimental procedures were approved by the Animal Ethics Committee of Sun Yat-sen University, Guangzhou, China (no. 2020000768). All animals received humane care according to the criteria outlined in the “Guide for the Care and Use of Laboratory Animals.”

### Eukaryotic expression and purification of recombinant SEPT2

PcDNA3.1-SEPT2 WT and PcDNA3.1-SEPT2 K74R vectors were transferred into HEK293F cell line by PEI (Proteintech, Rosemont, IL). Anti-FLAG-tag affinity purification kit (DIA-AN, Wuhan, China) was used to purify recombinant Flag-SEPT2 according to manufacturer’s instructions. In brief, anti-Flag affinity gel was added to HEK293F cell lysate (lysed with 0.3% Nonidet P40 IP lysis buffer) and incubated at 4 ℃ overnight. Proteins were then collected through centrifugation and were washed for five times with 0.5% Nonidet P40 IP lysis buffer. Recombinant SEPT2 was eluted with arginine-HCl (pH = 3.0) buffer, and pH was adjusted with 1 M Tris HCl buffer (pH = 8.0). Recombinant SEPT2 was concentrated in an Amicon^®^ Ultra4 tube (Merck Millipore, Germany).

### Phosphate determination with malachite green

Phosphate level was determined as previously reported [16]. In brief, 5 µl (50 µg) of recombinant protein and 10 µl of 4 mM GTP were added to 25 µl of assay buffer and incubated at room temperature for 15 min. 80 µl of color reagent was added to this mixture with incubation for 2 min. 10 µl of citrate solution was then added to each tube and incubated for 5 min. Absorbance was measured at A650 nm. Solutions: assay buffer (40 mM Tris, 80 mM NaCl, 8 mM MgAC2, 1 mM EDTA, pH = 7.5); A. Malachite green solution (0.05%); B. Ammonium molybdate (4.2%) in HCl; C. Citrate solution (34% of Na_3_Citrate•2H_2_O in di-H_2_O); D. 2% Nonidet P40 in di-H_2_O; E. Color reagent: 1 volume of “B” mixed with 3 volumes of “A” to which 0.1 ml of solution "D" in 5 ml was added.

### GST pull-down assay

pGEX-4t-1, pGEX-4t-1-SEPT2 WT and PGEX-4t-1-SEPT2K74R, PGEX-4t-1-SIRT2 plasmids were transformed into *E. coli* BL21. Protein prokaryotic expression was induced by 1 mM isopropyl-b-D-thiogalactoside (CWBIO, Jiangsu, China) at 37 °C for 4 h. Proteins were then purified using GST-tagged protein purification kit (Beyotime, Jiangsu, China). Purified proteins were mixed with anti-GST antibody-conjugated beads and cell lysate (lysed with 0.3% Nonidet P40 IP lysis buffer) or recombinant Flag-SEPT2 at 4 °C for 12 h. The beads were subsequently collected through centrifugation and were washed for five times with 0.5% Nonidet P40 IP lysis buffer before boiling with SDS-loading buffer and subjected to Western blot.

### Statistical analysis

Results are expressed as means ± standard deviation (SD). Statistical analysis and plotting were performed using SPSS statistical software package (version 18.0, IBM) and GraphPad Prism 7.0 software (GraphPad Software). In vitro experiments were repeated at least three times to achieve adequate power for detecting significant changes. One-way analysis of variance (ANOVA), Student’s t test and two-way ANOVA were performed to compare variations in groups in the functional assay. A p value less than 0.05 was considered statistically significant.

## Results

### The crotonylome was highly expressed in highly metastasis potential HCC tissues and cells

**Fig. 1:**
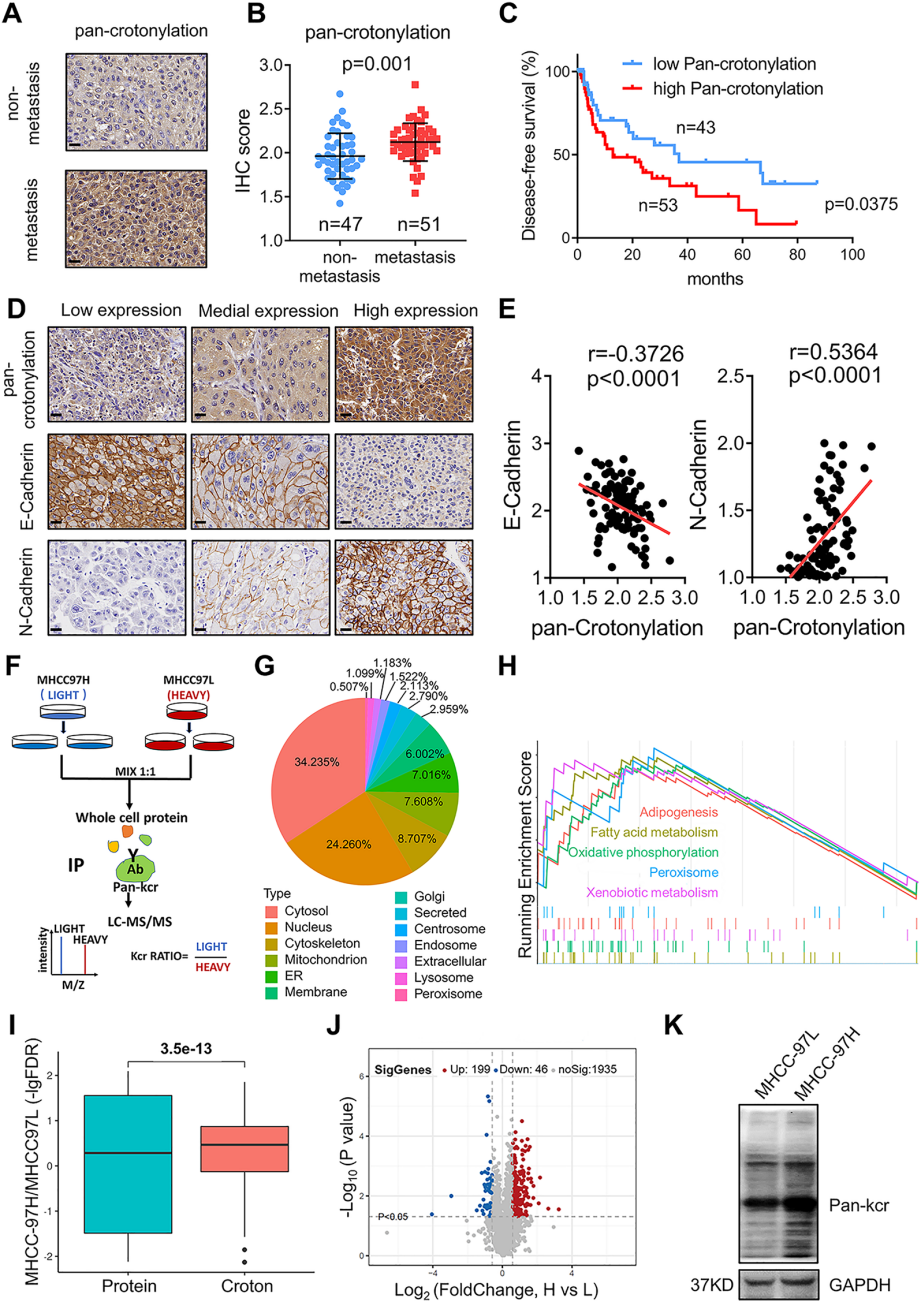
The crotonylome in HCC tissues and cells. **A**, **B** Immunohistochemistry (IHC) assays showing that pan-crotonylation was higher in HCC patients with metastasis. The data are presented as the means ± SD. (Student’s t test). **C** Patients with high pan-crotonylation levels had a shorter disease-free survival period than those with low pan-crotonylation levels. **D**, **E** IHC assays of 100 HCC tissues showing that pan-crotonylation was correlated with E-Cadherin and N-Cadherin. **F** Flowchart showing the experimental procedure followed for identifying crotonylated proteins through stable isotope labeling by amino acids in cell culture (SILAC) and liquid chromatography with tandem mass spectrometry (LC–MS/MS). **G** Subcellular localization of the identified crotonylated proteins. **H** Gene set enrichment analysis (GSEA) showing the five most enriched pathways (p < 0.05). **I** The difference in protein expression and crotonylation between MHCC-97H and MHCC-97L cells. The level of protein expression and degree of crotonylation were increased in MHCC-97H cells (-lg false discovery rate [FDR] > 0). **J** Scatter diagram showing the differentially crotonylated sites in the MHCC-97H and MHCC-97L cell lines (p < 0.05). **K** Western blotting (WB) verified that the total crotonylation level was higher in the MHCC-97H cell line

To explore the role of crotonylation in HCC metastasis, we detected the pan-crotonylation in 100 HCC tissues. We found higher pan-crotonylation in primary HCC tissues of patients with metastasis within one year after surgery (N = 51), compared to HCC patients without metastasis (N = 49) (p < 0.01) (Fig. 1A, B). Patients with higher crotonylation had shorter disease-free survival period (p < 0.05) (Fig. 1C). Besides, we observed that the crotonylation level were similar in HBV positive and negative HCC (Additional file 1: Fig. S1A). EMT is a key process of cancer cell metastasis. During this process, epithelial cells acquire the characteristics of mesenchymal cells to enhance cell mobility and their migration ability [17]. We therefore examined the correlation between crotonylation and EMT makers by immunohistochemistry (IHC) staining. Our results showed that pan-crotonylation was negatively correlated with E-cadherin (p < 0.0001) and positively correlated with N-cadherin (p < 0.0001), suggesting that crotonylation level was associated with metastasis in HCC (Fig. 1D, E).

To identify crotonylated proteins associated with tumor metastasis, two HCC cell lines MHCC-97L (minimally invasive) and MHCC-97H (highly invasive), which were isolated from one patient and showed differential invasive potential (Additional file 1: Fig. S1B) [18], were used to perform SILAC. Differentially crotonylated proteins (Additional file 2: Data S1) were identified by LC–MS/MS (Fig. 1F), including 1085 crotonylated proteins and 3540 crotonylation sites. The analysis on subcellular distribution of crotonylated proteins showed that 34.2% of crotonylated proteins were located in the cytosol, 24.2% in the nucleus, 8.7% in the cytoskeleton, and 7.6% in the mitochondrion (Fig. 1G). Crotonylated proteins were enriched in several metabolic pathways, including adipogenesis and fatty acid metabolism, oxidative phosphorylation, peroxisome, and xenobiotic metabolism pathways, compared to non-crotonylated proteins (Fig. 1H).

We next analyzed all protein sequences in modified 21-mers constituted with amino acids in specific positions (10 amino acids upstream and downstream of one site) (Additional file 1: Fig. S1C). The motif-X analysis showed that EKE, KE, and EKxE were overrepresented Kcr hotspot sites (Additional file 1: Fig. S1D). Further analysis on the differences between minimally and highly invasive cell proteomes showed that the levels of protein expression and crotonylation were generally increased in highly invasive cells, and the increase in crotonylation was more obvious than that in protein expression **(**Fig. 1I). When compared with minimally invasive cells, we identified 334 upregulated and 205 downregulated proteins (fold change [FC] > 1.5, p < 0.05) (Additional file 1: Fig. S1E), and 199 hypercrotonylated and 46 hypocrotonylated sites in highly invasive MHCC-97H cells (FC > 1.5, p < 0.05) (Fig. 1J). In addition, the total crotonylation level was significantly higher in highly invasive cells than in minimally invasive cells (p < 0.05) (Additional file 1: Fig. S1F), which were confirmed by Western blot (Fig. 1K). Collectively, our findings revealed the increase of crotonylation in HCC tissues with metastasis as well as highly invasive HCC cells.

### Crotonylation was positively correlated with HCC cell migration and invasion

**Fig. 2:**
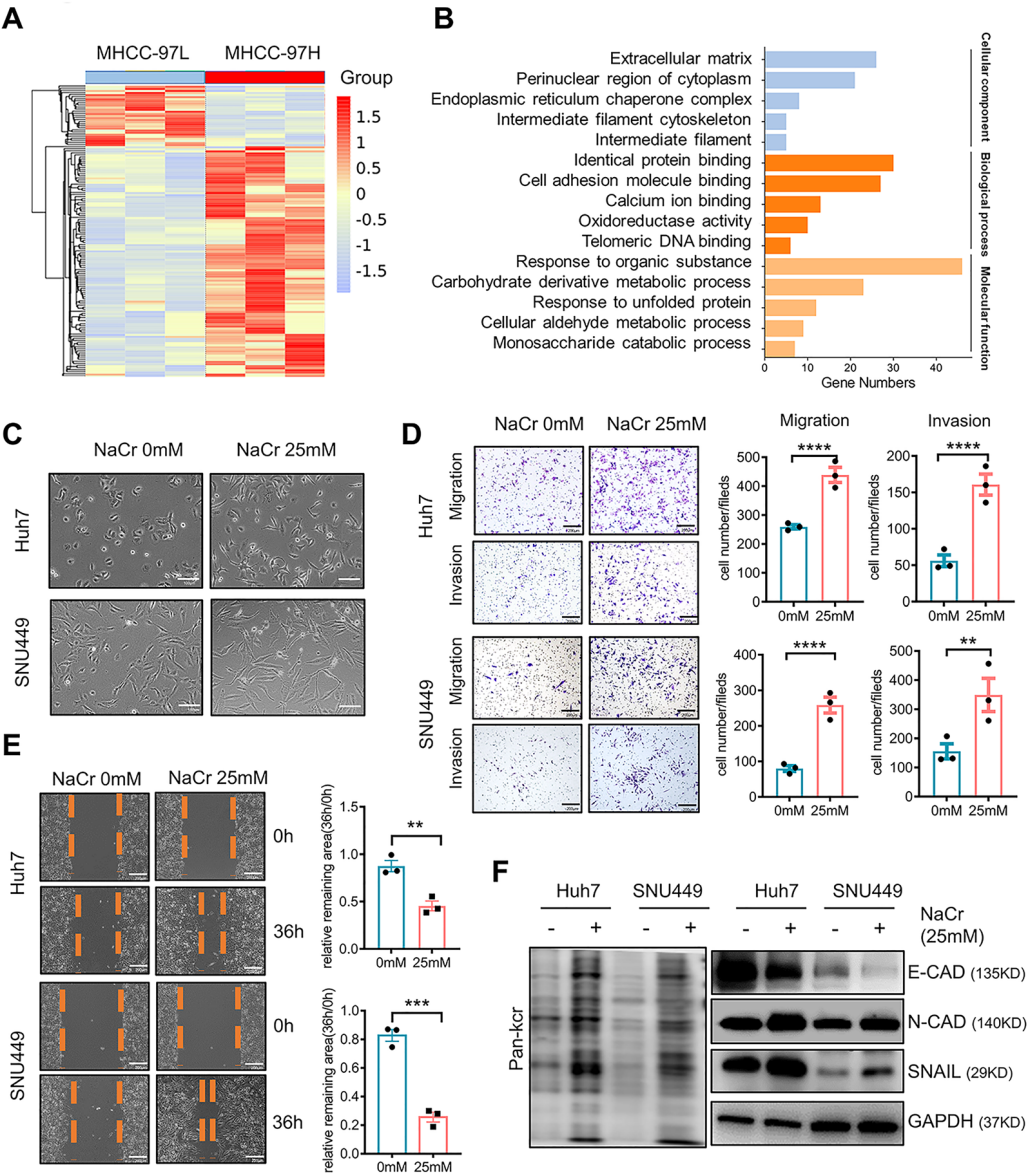
Crotonylation was positively correlated with HCC cell migration and invasion **A** Heatmap showing differentially crotonylated proteins in the MHCC-97H and MHCC-97L cell lines. **B** Gene ontology (GO)-based enrichment analysis of differentially crotonylated proteins. **C** Morphological changes in SNU449 and Huh7 cells after 25 mM sodium crotonate (NaCr) treatment. **D** Migration and invasion assays showing that SNU449 and Huh7 cells had higher invasive potential after 25 mM NaCr treatment. The data are presented as the means ± SD. **p < 0.001, ****p < 0.0001 (Student’s t test). **E** Wound healing assays showing that SNU449 and Huh7 cells exhibited greater migration capacity after 25 mM NaCr treatment. The data are presented as the means ± SD. **p < 0.01, ***p < 0.001 (Student’s t test). **F** Western blot analysis showing the changes in the expression of epithelial-mesenchymal transition (EMT)-related proteins after 25 mM NaCr treatment

To investigate the association of crotonylation with metastasis, we analyzed differentially crotonylated proteins between the two HCC cell lines. As shown in Fig. 2A, highly invasive MHCC-97H cells had more hypercrotonylated proteins. Gene set enrichment analysis showed that in highly invasive cells, more crotonylated proteins were enriched in biological oxidation and metabolism of xenobiotics by cytochrome P450 processes, with fewer enriched in cell junction organization and focal adhesion processes, compared with crotonylated proteins in minimally invasive cells (Additional file 1: Fig. S2A, B). Gene Ontology (GO) annotation revealed that hypercrotonylated proteins in highly invasive cells were involved in diverse biological processes, particularly the extracellular matrix and cell adhesion molecule binding (Fig. 2B). In addition, we found higher levels of crotonylation on known metastasis-related proteins in highly invasive cells (Additional file 1: Fig. S2C).

For verification, we increased the overall level of crotonylation in SNU449, Huh7 and SMMC7721 cell lines by adding crotonylation substrate (NaCr, sodium crotonate). An anti-pan-crotonyl-lysine (pan-Kcr) antibody was used to detect the total crotonylation level in cells after NaCr treatment. As shown in Additional file 1: Fig. S3, the total crotonylation reached the maximum after NaCr (25 mM) treatment. SNU449, Huh7 and SMMC7721 cells treated with NaCr exhibited an elongated spindle shape (Fig. 2C, Additional file 1: Fig. S4A). Increasing crotonylation by NaCr treatment promoted migration and invasion of SNU449, Huh7 and SMMC7721 cells (Fig. 2D, E, Additional file 1: Fig. S4B, C). Furthermore, increasing NaCr concentration decreased the expression of epithelial markers E-cadherin, while enhancing the expression of mesenchymal markers N-cadherin and Snail in HCC cells (Fig. 2F, Additional file 1: Fig. S4D). These results indicated that increasing crotonylation exerted a promoting effect on HCC cell migration and invasion.

### SEPT2 was identified in the crotonylome

**Fig. 3:**
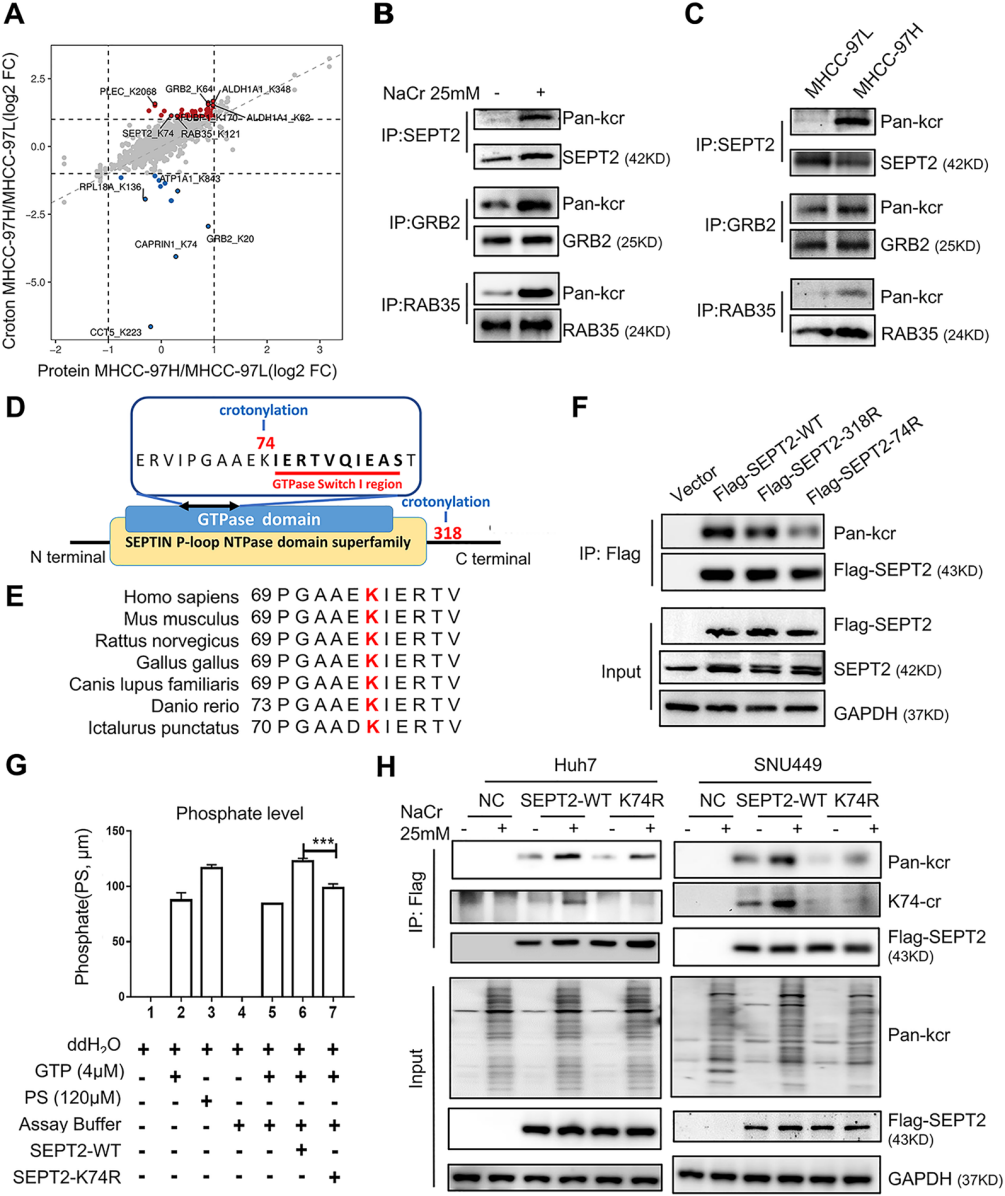
Lysine (K) 74 crotonylation of SEPT2 was identified. **A** Scatter diagram based on the fold change (FC) of protein expression (FC < 2) and crotonylation (FC > 1.5). **B** Validation of endogenous crotonylation of SEPT2, GRB2 and RAB35. MHCC-97L cells were treated with 25 mM sodium crotonate (NaCr) for 24 h. Cell lysates were immunoprecipitated with corresponding primary antibodies, followed by western blotting (WB) with an anti-pan-Kcr antibody. **C** Validation of endogenous differences in SEPT2, GRB2 and RAB35 crotonylation in MHCC-97H and MHCC-97L cell lines. SEPT2 crotonylation was highest crotonylation in the MHCC-97H cells. **D** The location of differentially crotonylated sites in the SEPT2 domain. **E** SEPT2-K74 is evolutionarily conserved in seven species. K74 in SEPT2 is highlighted in red. **F** The SEPT2-K74 and SEPT2-K318 mutants were crotonylated to a lesser extent than wild-type (WT) SEPT2. The degree of crotonylation in cells with ectopically expressed Flag-tagged SEPT2 WT, K74R and K318R was analyzed. **G** GTPase activity was impaired after SEPT2-K74R mutation. Flag-tagged SEPT2 WT and K74R proteins were purified, and an in vitro GTP hydrolysis assay was performed. Panels 1, 2, 4, and 5 show the negative controls, and panel 3 shows the positive control. **H** Western blot analysis of overexpressed Flag-tagged SEPT2 WT and K74R, pan-crotonylation and SEPT2 K74-crotonylation (K74-cr) in cells and crotonylation on SEPT2 with or without NaCr treatment

To explore the mechanism by which crotonylation facilitates cell migration and invasion in HCC, the proteomes of highly and minimally invasive cells were analyzed. After normalization, the 3 most significantly differential crotonylated proteins- SEPT2, RAB35, and GRB2, were selected (Fig. 3A, Additional file 1: Fig. S5A). After NaCr treatment, the results showed that these three candidates were hypercrotonylated in MHCC-97L cells (Fig. 3B). Furthermore, analysis on endogenous crotonylation revealed that the crotonylation level of these three proteins was greater in highly invasive cells compared to minimally invasive cells, and these results were consistent with SILAC analysis (Fig. 3C).

Among the 3 protein candidates, SEPT2 was eventually selected as it had the greatest level of crotonylation in highly invasive cells. SEPT2 is crucial in spindle formation and sister chromatid separation, thereby regulating G2/M phase transition and cell mitosis [19]. Whereas several studies have shown that SEPT2 can facilitate tumor growth and metastasis [20, 21]. We identified that the two crotonylation sites in SEPT2, K318 (H/L = 1.3, p < 0.05) and K74 (H/L = 2.19, p < 0.05), were significantly differentially crotonylated, as determined by LS-MS/MS. Analysis on the protein domains in SEPT2 showed that K74 site is in the GTPase domain, and K318 site is in the variable domain (Fig. 3D). Through analyzing the protein sequence alignment of SEPT2 homologs in different species, our results revealed that K74 was more evolutionarily conserved than K318 (Fig. 3E, Additional file 1: Fig. S5B). In addition, when the lysine residue at 74 site was replaced with an alanine, SEPT2 crotonylation was significantly reduced, largely abrogating SEPT2 crotonylation, whereas crotonylation of the K318R mutant was slightly reduced (Fig. 3F). These results indicated that K74cr may play a functional role in SEPT2. Therefore, we purified ectopically expressed SEPT2 wildtype (WT) and SEPT2-K74R in HEK293FT cells (Additional file 1: Fig. S5C) and performed GTPase assay. The results showed that SEPT2-K74R significantly impaired GTP degradation activity of SEPT2 (p < 0.001) (Fig. 3G). To better examine the role of SEPT2-K74cr, we generated a site-specific anti-SEPT2-K74 antibody that could target K74cr in SEPT2. The specificity of this antibody was confirmed, as the results of dot blot, and Western blot showed that K74cr preferentially detected SEPT-K74cr but not unmodified SEPT2 (Additional file 1: Fig. S5D, Fig. 3H).

### SEPT2-K74R mutant inhibited HCC cell migration and invasion in vitro and in vivo

**Fig. 4:**
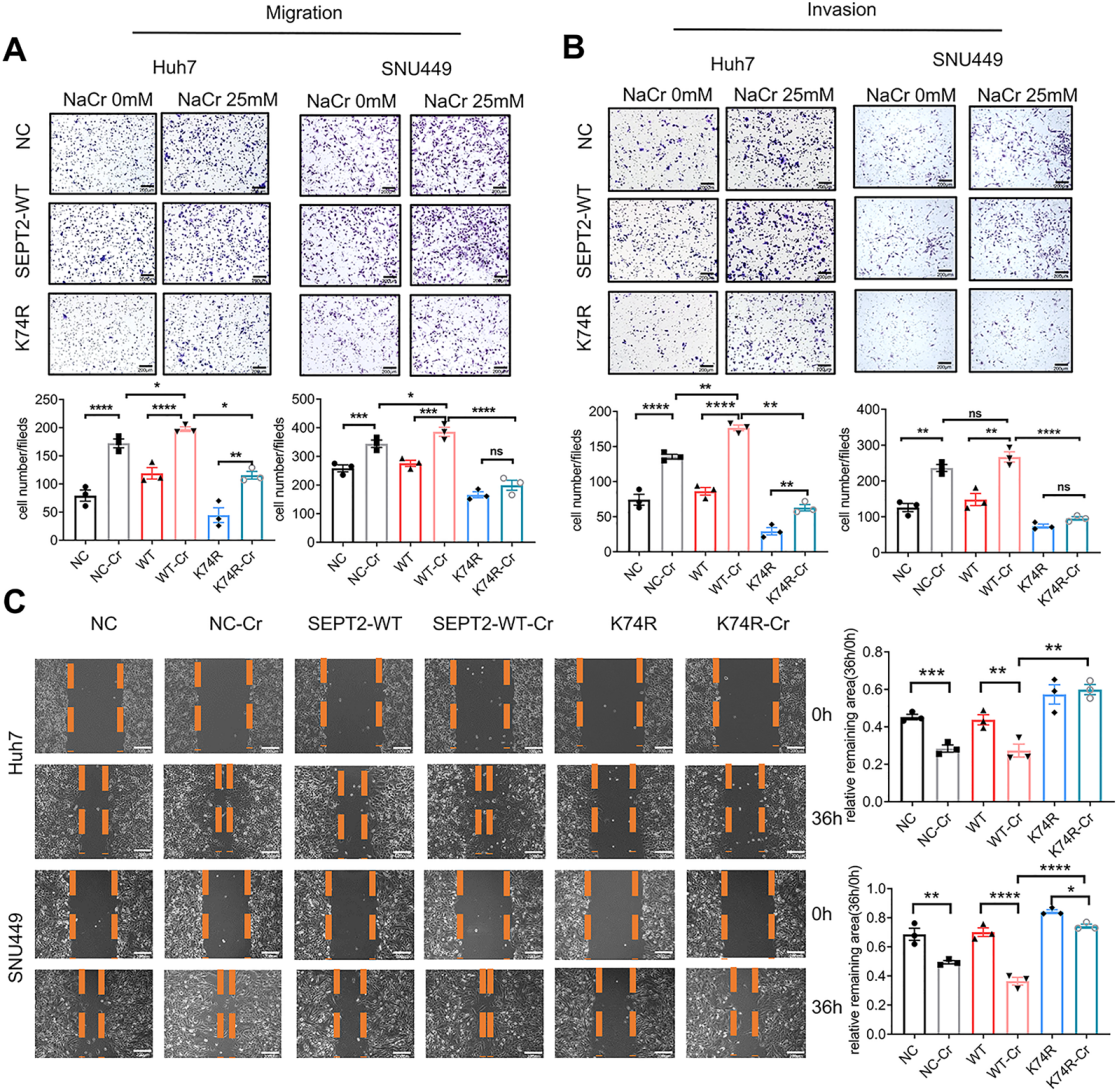
SEPT2-K74R inhibited cell migration and invasion in vitro. **A** Overexpression of SEPT2-K74R inhibited migration of Huh7 cells (left panel) and SNU449 cells (right panel) with and without 25 mM sodium crotonate (NaCr) treatment. **B** Overexpression of SEPT2-K74R inhibited the invasion of Huh7 cells (left panel) and SNU449 cells (right panel) with and without 25 mM NaCr treatment. **C** Overexpression of SEPT2-K74R inhibited the migration of Huh7 cells (upper panel) and SNU449 cells (lower panel) with and without 25 mM NaCr treatment. The data are presented as the means ± SD. Ns p > 0.05, *p < 0.05, **p < 0.01, ***p < 0.001, ****p < 0.0001 (one-way ANOVA). NC, negative control; WT, wild type; 74R, SEPT2-K74R; NC-Cr, SEPT2-WT-Cr and K74R-Cr indicate NC, SEPT2-WT and SEPT2-K74R cells treated with 25 mM NaCr, respectively

To investigate the functional role of SEPT2-K74 crotonylation, we stably overexpressed SEPT2-WT and SEPT2-K74R in SNU449, Huh7 and SMMC7721 cells. The forced expression of SEPT2-WT and SEPT2-K74R as well as whole-cell crotonylation and crotonylation of SEPT2 with or without NaCr treatment were verified by Western blot (Fig. 3, Additional file 1: Fig. S5E). Additional file 1: Fig. S5F showed that K74R could decrease the acetylation and succinylation of SEPT2, but NaCr treatment had no effect on SEPT2 acetylation and succinylation. We observed an increased metastatic potential of SEPT2-WT cells treated with NaCr compared to control cells. In contrast, migration and invasion of K74R-overexpressing cells with and without NaCr treatment were both inhibited, compared to SEPT2-WT and control cells (Fig. 4A–C, Additional file 1: Fig. S6A, B). These results thus implicated the anti-metastasis potential of SEPT2-K74R. Besides, we examined the impact of overexpressing SEPT2-WT and SEPT2-K74R on HCC cell growth and cell cycle with or without NaCr treatment. We found SEPT2-WT overexpressing has a positive effect on HCC cell growth, but K74R and NaCr treatment could hamper HCC cell growth (Additional file 1: Fig. S7A, B). Flow cytometry analysis of SEPT2-WT overexpressing cells, but not K74R, showed an increase in S phase and a reduction in G1 phase, and cells treated with NaCr showed a reduction of cells in S phase and an increase of cells in G1 phase (Additional file 1: Fig. S7C). It means that K74 decrotonylation could inhibit cell growth and cell cycle, but the role of NaCr inhibited cell growth and cycle may not due to SEPT2 crotonylation.

**Fig. 5:**
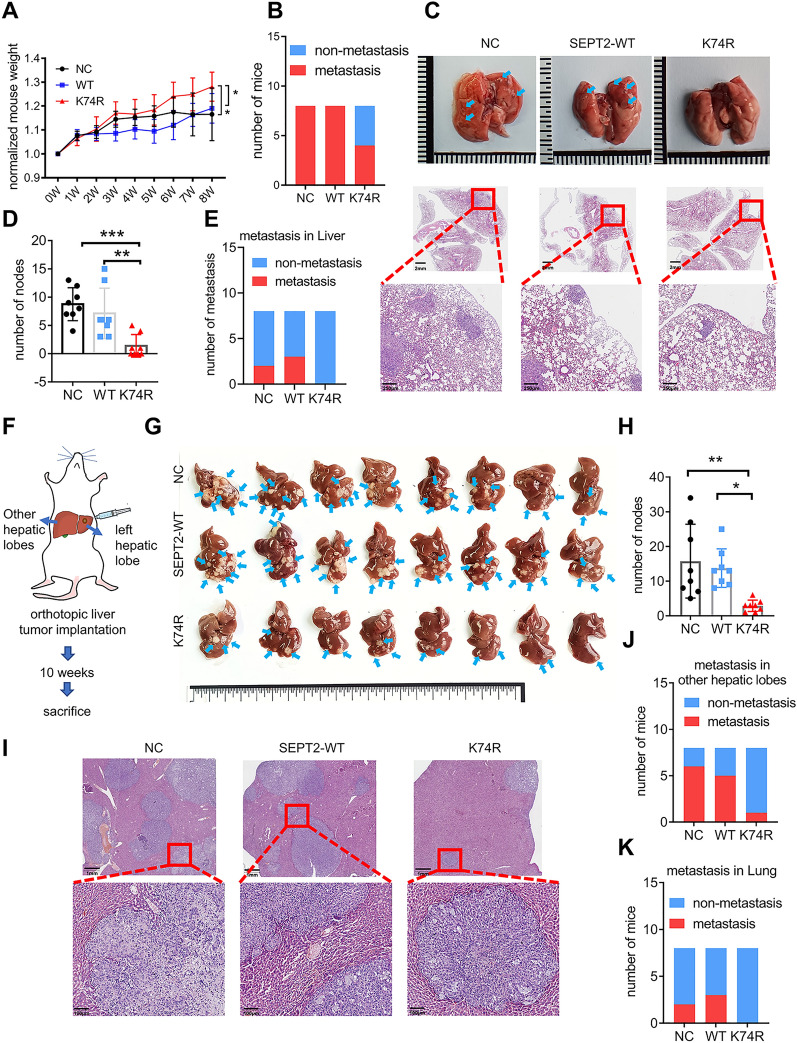
SEPT2-K74R inhibited cell migration and invasion in vivo **A** Mice in the SEPT2-K74R group gained the most weight. Huh7 stable cells were injected into the tail vein of NCG mice (8 mice each group). The mice were weighed every week (W) and were sacrificed 8 weeks after the injection. **B** Quantitative comparison of mice with lung metastases in each group. Fewer mice had lung metastasis in the SEPT2-K74R group. The red column indicates the mice had metastases. **C** Representative images of lung metastases in the tail vein injection model. After the mice were sacrificed, the lungs were dissected and photographed (upper panel). Blue arrows indicate lung metastases. Lung tissues were stained by H&E (bottom panel). **D** Quantitative comparison of the number of lung metastases in each group. **E** Quantitative comparison of mice with liver metastases in each group. The red column indicates the transferred mice. **F** Flowchart showing the orthotopic liver tumor implantation model. Huh7 stable cells were injected into the left lobe of the liver in NCG mice (8 mice per group). **G** Images of liver metastases in the orthotopic liver tumor implantation model. After the mice were sacrificed, the livers were dissected, photographed and weighed. Blue arrows indicate liver metastases. **H** Quantitative comparison of the number of liver metastases in each group. Mice in the SEPT2-K74R group had fewer liver metastases. **I** Representative images showing liver metastases in the orthotopic liver tumor implantation model. Tissues were stained by H&E. **J**, **K** Quantitative comparison of the number of mice with metastases in other lobes of the liver (**J**) and the number of mice with lung metastases (**K**). The red column indicates the transferred mice.The data are presented as the means ± SD. *p < 0.05, **p < 0.01, ***p < 0.001, ****p < 0.0001 (one-way ANOVA, two-way ANOVA). NC, negative control; WT, wild-type SEPT2; K74R, SEPT2-K74R mutant

To validate our in vitro findings, we established multiple mouse models with tail vein injection, orthotopic liver tumor implantation, and splenic vein injection. For mice with tail vein injection, Huh7 cells carrying a stable overexpression vector, SEPT2-WT, or SEPT2-K74R, were injected into the tail vein of NCG mice (N = 8 per group). Mice were sacrificed 8 weeks later, and HCC metastasis in the lungs was assessed (Additional file 1: Figure S8A). The results showed that mice in the SEPT2-K74R group were more nutrified (Additional file 1: Fig. S8B) with significantly higher body weight than the other two groups (both p < 0.05) (Fig. 5A). Mice in SEPT2-K74R (4/8) group also had fewer lung metastases than the SEPT2-WT (8/8) or vector (8/8) groups (Fig. 5B–D). In addition, we observed liver metastases in the vector (2/8) and SEPT2-WT (3/8) groups but not in the SEPT2-K74R group (Fig. 5E, Additional file 1: Fig. S8C).

We then established an orthotopic mouse model with liver tumor implantation by injecting Huh7 cells into the left lobe of liver, and mice were sacrificed 10 weeks later (Fig. 5F). The relative liver weight in mice with K74R was significantly lower than the vector group (p < 0.05) (Additional file 1: Fig. S9A). We observed significantly fewer metastases in the left lobe of liver (Additional file 1: Fig. S9B) and the total liver (Fig. 5G, I) in mice with K74R than in the other two groups. Fewer metastases in other liver lobes (1/8) and lung (0/8) in the K74R group were also observed, compared to the vector group (6/8 with liver and 2/8 with lung metastases) and SEPT2-WT group (5/8 with liver and 3/8 with lung metastases) (Fig. 5J, K, Additional file 1: Fig. S9C). In addition, a further mouse model was established by injecting Huh7 cells into the spleen, and mice were sacrificed 8 weeks later (Additional file 1: Fig. S10A). Consistent with the findings of other mouse models, the K74 group had lower incidence of intrahepatic metastases, compared to the vector and SEPT2-WT groups (Additional file 1: Fig. S10B–E). Altogether, both in vitro and in vivo results illustrated that SEPT2-K74R mutant could markedly inhibit HCC metastasis.

### SIRT2 decrotonylated SEPT2

We co-expressed SEPT2-WT with four decrotonylases, SIRT1, SIRT2, SIRT3 and HDAC3, which were verified in a previous study [8] in 293 T cells (Additional file 1: Fig. S11A). Notably, only SIRT2 had the ability to decrotonylate SEPT2, while the other three decrotonylases exerted no effect on SEPT2 Kcr. For verification, we performed co-IP assay (Additional file 1: Fig. S11B) and GST pull-down assay (Additional file 1: Fig. S11C), and both results confirmed that SIRT2 could interact with SEPT2. Furthermore, SIRT2 decronylated SEPT2 in a dose-dependent manner (Additional file 1: Fig. S11D), and knockdown of SIRT2 expression and SIRT2 inhibitor, AGK2, treatment led to a significant increase in SEPT2 crotonylation (Additional file 1: Fig. S11E, F). Therefore, our results indicated that SIRT2 could decrotonylate SEPT2. Moreover, we co-expressed SEPT2-WT with four crotonylases [10], CBP, P300, HMOF and PCAF in 293 T cells (Additional file 1: Fig. S11G). The result showed that CBP and P300 could interact with SEPT2 and had the ability to crotonylate SEPT2, while others exerted no effect on SEPT2 crotonylation.

### Crotonylation promoted cell invasive capability mediated through the SEPT2-K74cr-P85α-Akt pathway

**Fig. 6:**
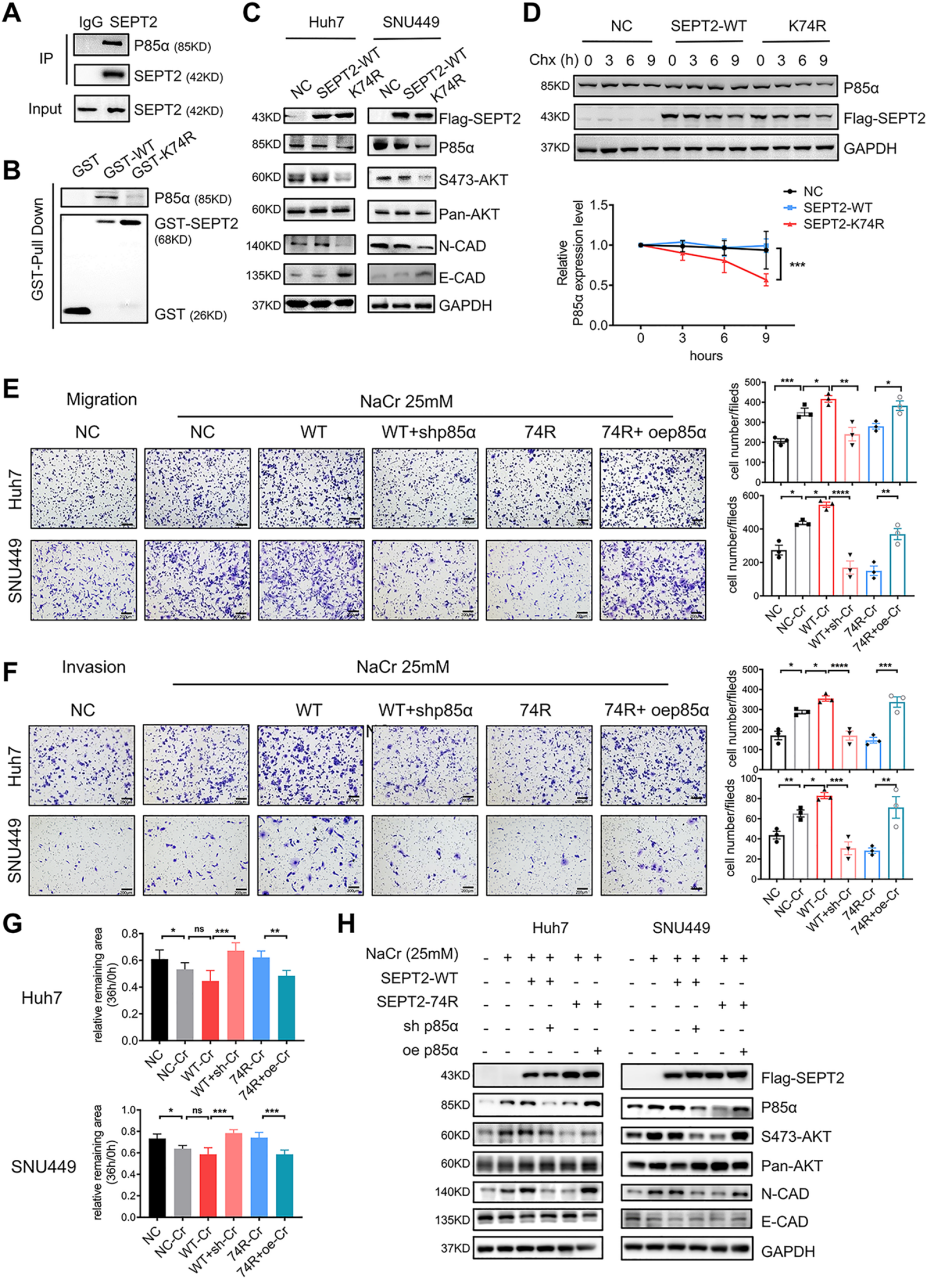
Crotonylation enhanced cell invasive capability by mediating SEPT2-K74Cr-P85α-Akt pathway activity (**A**, **B**) P85α interacts with SEPT2. Whole-cell lysates were immunoprecipitated with an anti-SEPT2 antibody (**A**) and GST-tagged SEPT2-WT or GST-tagged SEPT2-K74R were purified with anti-GST antibody-conjugated beads **B**. **C** SEPT2-K74R overexpression decreased the expression of downstream P85α. **D** SEPT2-K74R overexpression decreased P85α stability. Transient overexpression vectors carrying SEPT2 WT and SEPT2-K74R in SNU449 cells were treated with cycloheximide (CHX), and the P85α protein level was determined by WB (upper panel). The lower panel shows the relative protein levels in the different groups. Error bars represent ± SD on the basis of triplicate experiments. **E**, **F** P85α rescued SEPT2 function after sodium crotonate (NaCr) treatment. Knocking down P85α expression inhibited cell migration (E) and invasion (**F**) after NaCr treatment; overexpression of P85α in SEPT2-K74R-expressing cells rescued the cell migration (**E**) and invasion (**F**) abilities after NaCr treatment. **G** P85α rescued SEPT2 function after NaCr treatment. Quantitative comparison of wound-healing assays performed with Huh7 cells (upper panel) and SNU449 cells (lower panel). **H** Western blot analysis of proteins expressed in the P85α rescue assays.The data are presented as the means ± SD. *p < 0.05, **p < 0.01, ***p < 0.001, ****p < 0.0001 (one-way ANOVA, two-way ANOVA). NC, negative control; WT, wild-type SEPT2; K74R, SEPT2-K74R mutant; Cr, NaCr treatment at25 mM; sh, short interfering RNA downregulation of p85α expression; oe, p85α overexpression

SEPT2 plays an important role in mitosis as it is crucially involved in spindle formation and sister chromatid separation. A previous study showed that P85α, a subunit of PI3K which is important for EMT [22, 23], interacted with SEPT2 to regulate mitosis and that SEPT2 GTPase activity is crucial for mitosis [24]. Therefore, we hypothesized that P85α may be related to SEPT2-K74cr, and P85α. By co-IP and GST pull-down assays, our results found that K74R mutation impaired the interaction of SEPT2 with P85α (Fig. 6A, B). A reduction of P85α expression in SEPT2-K74R-overexpressing cells was observed (Fig. 6C). While the mRNA expression of P85α did not change in SNU449 cells and even increased in Huh7 cells (Additional file 1: Fig. S12A), indicating that SEPT2-K74R regulated P85α protein level mainly at posttranscriptional level. For verification, SEPT2-74R was transiently overexpressed in SNU449 cells, and these cells were treated with the protein synthetase inhibitor CHX in FBS-free medium. The results showed that P85α protein level was decreased in SEPT2-K74R-overexpressing cells treated with CHX (p < 0.001) (Fig. 6D). In addition, we observed a decrease in the stability of P85α in SIRT2-overexpressing cells (Additional file 1: Fig. S12B).

To identify which the protein degradation pathway K74R mutant regulates P85α level, SNU449 cells with transient SEPT2-74R overexpression were treated with the proteasome inhibitor MG132 and autophagy inhibitor Baf1A. The results showed that MG132 exerted no effect on P85α degradation, while Baf1A slowed P85α degradation in CHX-treated cells, indicating that SEPT2-K74R could enhance P85α stability by influencing its autophagic degradation (Additional file 1: Fig. S12C). Since P85α is a key protein in the PI3K-Akt pathway, we examined the interplay between this pathway and SEPT2-K74R. The results showed that PI3K-Akt pathway activities in SEPT2-K74R cells and SEPT2-K74R mouse tumor tissues were significantly decreased, and we observed a reduced expression of N-cadherin and an increased expression of E-cadherin (Fig. 6C, Additional file 1: Fig. S12D). Furthermore, knocking down P85α in conjunction with NaCr treatment inhibited the invasive capability of SEPT2-WT cells, while this trend was reversed by overexpressing P85α in SEPT2-K74R cells (Fig. 6E, F, Additional file 1: Fig. S13A, B). We also observed that P85α was overexpressed in SEPT2-WT cells after NaCr treatment but not in P85α-knockdown or SEPT2-K74R-overexpressing cells (Fig. 6G). These results collectively indicated that crotonylation could promote EMT through the SEPT2-K74Cr-P85α-AKT pathway.

### Crotonylation of SEPT2 was associated with poor prognosis and recurrence in HCC patients

**Fig. 7:**
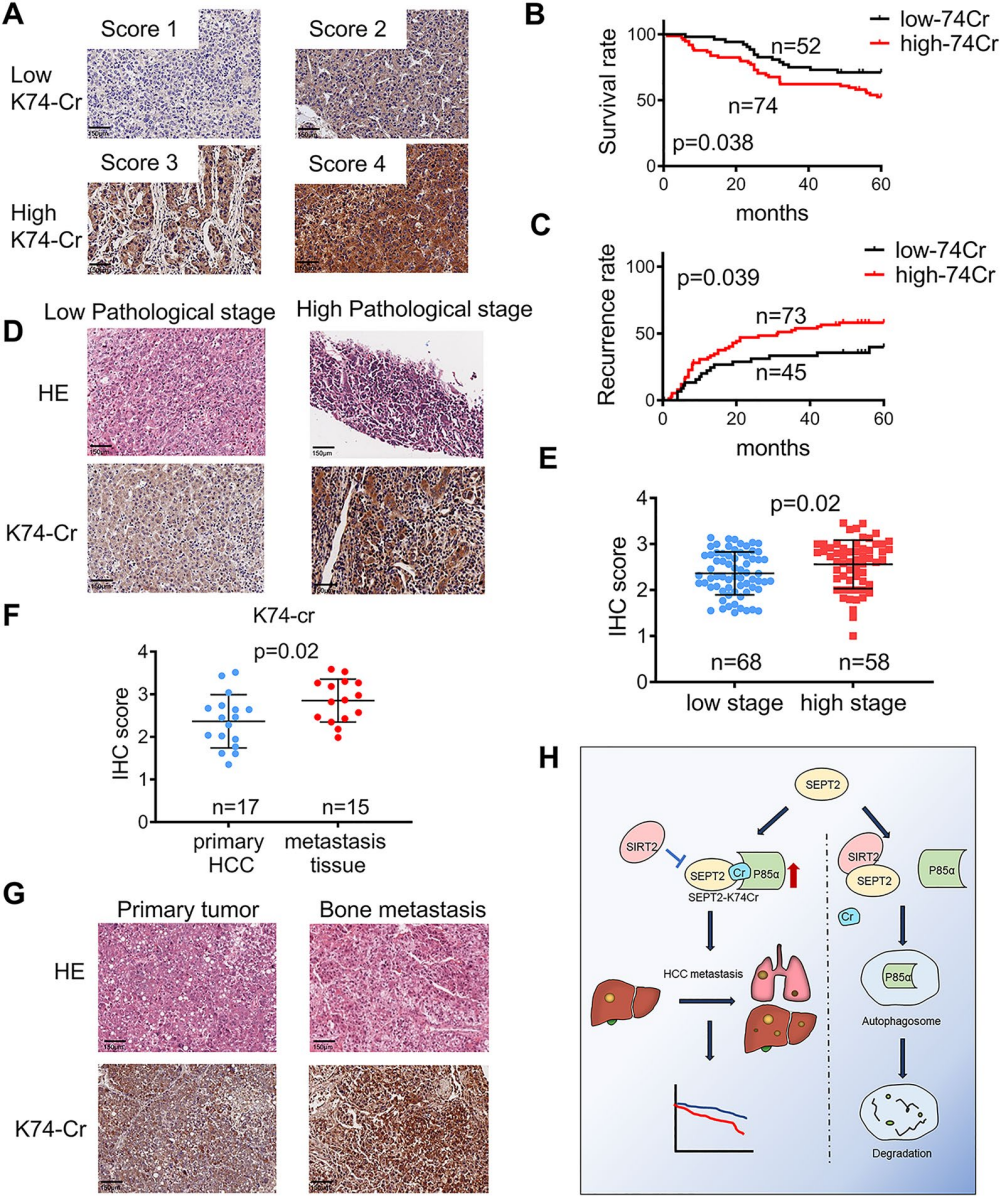
SEPT2-K74cr may be a potential biomarker for predicting prognosis and HCC recurrence in patients (**A**) Scores indicate crotonylation of SEPT2-K74cr (K74-Cr) in representative tumor tissues as determined by immunohistochemistry (IHC) staining. Scores were calculated on the basis of the staining intensity and percentage of stained cells. **B** Patients with high K74-Cr levels had lower overall survival than those with low K74-Cr levels. **C** Patients with high K74-Cr levels had a higher recurrence rate than those with low K74-Cr levels. **D** Representative images of tissues with low and high stage pathology as determined by hemoxylin and eosin (**H**, **E**) and IHC staining. **E** Quantitative comparison of the IHC score in the tissues with low and high stage pathology. The data are presented as the means ± SD. *p < 0.05. **F** Quantitative comparison of the K74-Cr IHC scores in metastasis tissue and primary HCCs in Tissue microarray (HLivH060CD03) (**G**) Images showing bone metastasis tissue and the corresponding primary HCC tumor after H&E and IHC staining. **H** Graphic model showing the effect of SEPT2-K74 crotonylation in HCC. K74 crotonylation was negatively regulated by SIRT2, and K74 decrotonylation decreased P85α stability and PI3K pathway activity, which inhibited HCC metastasis. SEPT2-K74 crotonylation can serve as an independent predictor of low patient survival and HCC recurrence

To examine the clinical importance of SEPT2-K74cr in HCC patients, we use the site-specific anti-SEPT2-K74 antibody to do IHC assays in HCC samples. The specificity of this antibody was confirmed by IHC in adherent cells (Additional file 1: Fig. S14A). A total of 126 HCC samples were collected for IHC analysis. Based on the percentage of positively stained cells and color intensity, each sample was given a score of 1 to 4 for SEPT2-K74cr level (Fig. 7A), and the score was then correlated with different clinical parameters. We found that HCC patients with low SEPT2-K74cr level had better prognosis (p < 0.05) (Fig. 7B) and lower recurrence rate (p < 0.05) (Fig. 7C). When correlating with cancer stages, SEPT2-K74cr protein level was found to be higher in patients with early HCC (stage I/II) than in patients with advanced HCC (stage II/III) (p < 0.05) (Fig. 7D, E). Moreover, we observed a significant increase (p < 0.05) of the SEPT2-K74cr level (Fig. 7F) in metastatic HCC tumors compared with primary HCC, but not pan-Kcr (Additional file 1: Fig. S14B). Figure 7G showed a higher SEPT2-K74cr level in bone metastasis tissue compared to its corresponding primary HCC tumor.

We further assessed the association of SEPT2-K74cr with HCC prognosis. The distribution of patients’ characteristics based on survival and recurrence status were showed in Additional file 1: Table S1 and S2, respectively. By univariate Cox regression analysis (Table 1), BCLC stage and SEPT2-K74cr level were found to be significant prognostic factors of HCC (p < 0.05), whereas low SEPT2-K74cr level was associated with reduced risk of cancer-related death (relative risk [RR], 0.536; 95% confidence interval [CI] 0.293–0.982; p < 0.05). Moreover, by multivariate Cox regression analysis, the SEPT2-K74cr level was found to be an independent predictor for low survival (RR, 0.521; 95% CI 0.285–0.955; p < 0.05) and recurrence in HCC patients (RR, 0.575; 95% CI 0.330–0.999; p = 0.05) (Table 1 and Additional file 1: Tables S1–S3). Altogether, our findings demonstrated that SEPT2-K74cr could be a potential prognostic factor for HCC patients.

**Table 1:** Cox Regression Analysis of Potential Poor Prognostic Factors for HCC Patients

	Variable		RR(95% confidence interval)	P value
Univariate cox regression	BCLC stage	Low stage	0.487(0.275–0.864)	0.014
		High stage	1.00	
	Age		1.015(0.991–1.041)	0.224
	Gender	MALE	1.279(0.504–3.200)	0.612
		FEMALE	1.00	
	SEPT2-K74cr level	Low expression	0.536(0.293–0.982)	0.044
		High expression	1.00	
Multivariate cox regression	BCLC stage	Low stage	0.474(0.267–0.841)	0.011
		High stage	1.00	
	SEPT2-K74cr level	Low expression	0.521(0.285–0.955)	0.035
		High expression	1.00	

## Discussion

In this study, by analyzing 100 HCC tissues and performing SILAC in two HCC cell lines with differential invasive potential, we found crotonylation was positively correlated with EMT process. Crotonylation is a very dynamic process which is largely dependent on its substrate. Previous study [25] showed that TSA, a HDACs inhibitor, and knock down HDACs could increases total lysine crotonylation level, and meanwhile inhibited cell migration. Reports have shown that crotonylation and acetylation share the same family of writers and erasers [8, 9]. HDACs have been reported as deacylase, but are not specific for decrotonylation. It is undeniable that TSA and HDACs knockdown have broad biological functions and can affect several kinds of lysine posttranslational modification. NaCr can be synthesized into crotonyl-CoA, the direct substrate of crotonylation, by acyl-CoA synthetase short chain family member 2 (ACSS2) [26], hence NaCr can specifically enhance protein crotonylation without affecting acetylation [11]. Previous studies found that NaCr exhibits beneficial effects on acute kidney injury [27], and it can affect the cell cycle [11] and promote endoderm differentiation of human embryonic stem cells [28]. Here, we used NaCr to specifically increase overall crotonylation in cells and found that higher overall crotonylation level facilitated EMT and increased migration and invasion of HCC cells. Our results therefore demonstrated the metastasis-promoting role of crotonylation in HCC.

Crotonylation happens on proteins, including histone and non-histone. Histone crotonylation, belongs to epigenetics, regulates genes’ expression which plays important roles in multiple cellular functions, including spermatogenesis [7, 29], kidney injury [27] and signal-dependent gene activation [9, 30]. Because of the functionally diversity of non-histone proteins, the function of crotonylation on non-histone proteins is complex. Lysine crotonylation of DgTIL1 enhancing DgTIL1 protein stability, therefore modulates cold tolerance in chrysanthemum [31]. In colorectal cancer, upregulation of enolase (ENO1) crotonylation promotes cell metastasis [14]. Crotonylation of RPA1 mediated DNA repair by enhancing the interaction of RPA1 with ssDNA and/or HR factors [31]. In our study, crotonylation on K74-SEPT2 regulates the GTPase activity of SEPT2 and facilitates HCC metastasis. Unlike histone crotonylation, the role of crotonylation on non-histone proteins differs from proteins to proteins. Both histone and non-histone protein crotonylation are important. Their existence had greatly increased biological diversity and enhanced the adaptability of organisms to the changeable environment.

In addition to its precursor NaCr, crotonyl-CoA is an intermediate metabolite in fatty acid oxidation and lysine/tryptophan degradation. Acyl-CoA dehydrogenase short chain (ACADS) and acyl-CoA oxidase (ACOX) can metabolize butyryl-COA to crotonyl-CoA [28]. Metabolic abnormalities, such as butyryl-COA accumulation and ACADS or ACOX dysfunction, could result in intracellular crotonylation fluctuation. Given the close association of crotonylation with metabolism, exploring the mechanisms of how crotonylation is altered in HCC and whether such change is related to metabolic pathways, is of great interest in future studies.

We demonstrated that SEPT2 was hypercrotonylated in highly invasive cells. SEPT2 is crucial for cytokinesis, and the rate of SEPT2 GTP hydrolysis is indispensable for fiber distribution [22, 32]. We identified that SEPT2-K74 crotonylation affects SEPT2 GTPase activity, and a crotonylation-abrogating mutation of K74 could inhibit HCC metastasis in vitro and in vivo. Moreover, increase in the invasiveness of SEPT2-WT-overexpressing cells was observed after NaCr treatment compared to controls. This inferred that SEPT2 GTPase activity not only regulates mitosis but is also associated with tumor invasion and migration, and the crotonylation on SEPT2-K74 may shift the role played by SEPT2 between tumor promoter and inhibitor. Thus, modulating crotonylation of SEPT2-K74 may be a potential therapeutic strategy for the prevention and treatment of HCC.

A recent study showed that dynamic crotonylation of EB1-regulated accurate microtubule dynamics in mitosis [12], thus ensuring that vertebrate cells divide in the correct orientation. EB1 has been found to compete with SEPT2 for binding to guanosine’ 5’-O-[γ-thio] triphosphate (GTPγS)-stabilized microtubules [33]. As shown in Fig. 1C, we observed that 8.7% of crotonylated proteins were located in the cytoskeleton, which is higher than the 1% reported in previous studies [10]. Combined with our results from GO analysis, these findings showed that crotonylated proteins are enriched in intermediate filaments of the cytoskeleton. While a previous study reported that histone crotonylation regulates meiosis [32], our results indicated the important role of crotonylation in mitosis.

Postoperative recurrence and high malignancy confound HCC treatment; therefore, finding prognostic biomarkers of HCC is an urgent undertaking. PTMs have been widely studied as predictors of tumor prognosis; for example, glycosylation in tumors is a target for liquid biopsy sampling [34]. In comparison, crotonylation on this area is still largely unknown. In our study, by using a site-specific antibody, we found that crotonylation of SEPT2-K74 is higher in patients with HCC recurrence and associated with poor prognosis, suggesting SEPT2-K74cr can be utilized as a potential recurrence and prognostic predictor in HCC. Our study is the first to elucidate the role played by crotonylation as a tumor biomarker.

In conclusion, our study revealed a novel role of crotonylation in promoting HCC metastasis and invasion. Crotonylation-modulated SEPT2 enhanced cell invasive capability by elevating the activity of P85α-Akt pathway. High crotonylation of SEPT2-K74 could predict HCC patients with poor prognosis and high recurrence rate.

## Supplementary Information

**Additional file 1: Figure S1.** Crotonylome in HCC cells (A)IHC analysis showed the crotonylation level are similar in HBV positive and negative HCC. (B) Migration and invasion assays showing that MHCC-97H cells had higher invasive potential than MHCC-97L cells. The data are presented as the means ± SD. *p < 0.05, **p < 0.01 (Student’s t test). (C-D) Motif analysis of all identified crotonylated sites. (E)Scatter diagram showing differentially expressed proteins in the MHCC-97H and MHCC-97L cell lines (p<0.05). (F) The total intensity of crotonylation was higher in MHCC-97H cell line (p<0.05). **Figure S2.** Crotonylome in differential invasive HCC cell lines (A-B) KEGG analysis: (A) Reactome analysis (B) differential crotonylated proteins. (C) Heatmap of known metastatic-related proteins in MHCC-97H and MHCC-97L cell lines. **Figure S3.** WB analysis of total crotonylation of HCC cell lines after NaCr treatment. **Figure S4.** Crotonylation was positively correlated with HCC cell migration and invasion (A)Cell Morphological changes in SMMC7721 cell line after 25mM of NaCr treatment. (B) Migration and Invasion assays showed SMMC7721 cells had higher invasive potential after 25mM of NaCr treatment. Data presented as mean ± SD. *p < 0.01, **p<0.001. (Student’s t test). (C) Wound heal assays showed SMMC7721 cells had greater migration capacity after 25mM of NaCr treatment. Data presented as mean ± SD. *p < 0.01. (Student’s t test). (D) WB analysis of the changes in the expression of EMT-related proteins after 25mM of NaCr treatment. **Figure S5.** Lysine 74 crotonylation of SEPT2 was identified (A)Scatter diagram based on the false discovery rate (FDR) of protein expression and crotonylation. (B) SEPT2 K318 is less evolutionarily conserved in seven species compared with K74. K318 of SEPT2 was highlighted in red. (C) Coomassie blue of purified Flag-tagged SEPT2. (D) Dot blotting assay of site-specific antibody of SEPT2-K74 crotonylation (K74Cr). (E)WB analysis of overexpression of Flag-tagged SEPT2-WT and K74R, pan-crotonylation in cells and crotonylation on SEPT2 with or without NaCr treatment. (F) WB analysis of SEPT2 acetylation (Kac) and succinylation (Ksucc) in cells with or without NaCr treatment. **Figure S6.** SEPT2-K74R Inhibited Cell Migration and Invasion in vitro (A-B) Overexpression of SEPT2-K74R inhibited the ability of cell migration and invasion in SMMC7721 cells with and without 25mM of NaCr treatment. Data presented as mean ± SD. *p < 0.05, **p < 0.01, ***p < 0.001, ****p<0.0001 (One-way ANOVA). **Figure S7.** SEPT2-K74R and NaCr Inhibited Cell Oroliferation and Cell Cycle. CCK8 analysis (A), colony formation assay (B) and cell cycle assay (C) of SEPT2-WT and SEPT2-K74R overexpression cells with and without NaCr treatment. **Figure S8.** SEPT2-K74R Inhibited Cell Migration and Invasion in tail vein injection mode (A)Flowchart of the tail vein injection model. Huh7 stable cells were injected into the tail vein of NCG mice (8 mice each group). Mice were sacrificed 8 weeks after injection. (B) Mice in SEPT2-K74R group had better nutrition. (C) Representative images of liver metastases in tail vein injection model. **Figure S9.** SEPT2-K74R Inhibited Cell Migration and Invasion in orthotopic liver tumor implantation mouse model (A)Quantitative comparison of tumor burden by calculating liver weight to body weight ratio of each mouse in orthotopic liver tumor implantation mouse model. (B) Quantitative comparison of the number of liver metastases in left lobe. Mice in the SEPT2-K74R group had fewer liver metastasis in orthotopic liver tumor implantation mouse model. (C) Representative images of lung metastases in orthotopic liver tumor implantation mouse model. Data presented as mean ± SD. *p < 0.05, **p < 0.01, ***p < 0.001, ****p<0.0001 (One-way ANOVA). **Figure S10.** SEPT2-K74R Inhibited Cell Migration and Invasion in splenic vein injection mouse model (A)Flowchart of the splenic vein injection mouse model. Huh7 stable cells were injected into the tail vein of NCG mice (7 mice each group). Mice were sacrificed 8 weeks after injection. (B) Representative images of liver metastases in the splenic vein injection mouse model. Blue arrows showed the liver metastases. (C) Representative images of liver metastases in the splenic vein injection mouse model. Tissues were stained by HE. (D) Quantitative comparison of tumor burden by calculating liver weight to bod weight ratio of each mouse in the splenic vein injection mouse model. (E) Quantitative comparison of the number of liver metastases in each group. Mice in the SEPT2-K74R group had fewer liver metastasis. Data presented as mean ± SD. *p < 0.05 (One-way ANOVA). **Figure S11.** SIRT2 decrotonylated SEPT2. (A)SIRT2 decreased crotonylation of SEPT2. Whole-cell lysates were immunoprecipitated with anti-Flag antibody, and precipitated proteins were detected by anti-Flag and anti-pan-kcr antibodies. (B) SIRT2 interacts with SEPT2. Whole-cell lysates were immunoprecipitated with control IgG and anti-Flag antibody, and precipitated proteins were detected by anti-SEPT2 and anti-Flag-SIRT2 antibodies. (C) SIRT2 interacts with SEPT2 in vitro. Purified GST-tagged SIRT2 protein were immunoprecipitated with Purified Flag-tagged SEPT2 and GST antibody-conjugated beads. (D-E) WB analysis of crotonylation of SEPT2. SEPT2 crotonylation was detected with different amount of SIRT2 (D) and SIRT2 interference (E). (F) WB analysis of crotonylation of SEPT2 after AGK2 treatment. (G)CBP and P300 interact with SEPT2 and could crotonylate SEPT2. **Figure S12.** SEPT2-K74R downregulated P85α (A) qRT-PCR analysis of relative fold change of P85α mRNA expression in NC, SEPT2-WT (WT) and SEPT2-K74R (K74R) groups. Data presented as mean ± SD. *p < 0.05, **p < 0.01, ***p < 0.001, ****p<0.0001 (One-way ANOVA). (B) SIRT2 overexpression decreased P85α stability. SNU449 cells were treated with CHX and P85α protein was determined by WB (up panel). The down panel showed relative protein level of different groups. Error bars represent ±SD of triplicate experiments. T. *p < 0.05, **p < 0.01, ***p < 0.001, ****p<0.0001 (One-way ANOVA). NC, negative control. (C) BafA1 increased P85α stability under SEPT2-K74R overexpression. (D) WB analysis of the expression levels of NCAD, ECAD, P85α, Phospho-AKT （S473-AKT）, AKT in mouse tumor tissues. **Figure S13.** Crotonylation facilitated cell invasive capability in SEPT2-K74Cr -P85α-Akt pathway (A-B) P85α rescued the function of SEPT2 under NaCr treatment. Knock down of P85α inhibited cell migration under NaCr treatment; Overexpression of P85α in SEPT2-K74R rescued the ability of cell migration in Huh7 (A) and SNU449 (B) cells. **Figure S14.** (A) IHC assays in adherent SNU449 cells. We used IHC staining to examine the K74-cr level in stably overexpressed SEPT2 WT and SEPT2-K74R adherent HCC cells, as positive and negative control respectively. (B) Quantitative comparison of the Pan-Kcr IHC scores in metastasis tissue and primary HCCs in Tissue microarray (HLivH060CD03). **Table S1.** Distribution of Patients characteristics by survival status. **Table S2.** Distribution of Patients characteristics by recurrence status. **Table S3.** Univariate Cox Regression Analysis of Potential Recurrence Factors for HCC Patients.

**Additional file 2: Data S1.** Crotonylome data.

## Data Availability

The datasets used and/or analysed during the current study are available from the corresponding author on reasonable request.
