# Supplementary material for: SEPT2 crotonylation promotes metastasis and recurrence in hepatocellular carcinoma and is associated with poor survival
Source: Cell Biosci. 2023 Mar 22;13:63. doi: 10.1186/s13578-023-00996-7 (PMC10032003; doi:10.1186/s13578-023-00996-7)
Supplement: Supplementary file 1 — Additional file 1: Figure S1. Crotonylome in HCC cells (A)IHC analysis showed the crotonylation level are similar in HBV positive and negative HCC. (B) Migration and invasion assays showing that MHCC-97H cells had higher invasive potential than MHCC-97L cells. The data are presented as the means ± SD. *p < 0.05, **p < 0.01 (Student’s t test). (C-D) Motif analysis of all identified crotonylated sites. (E)Scatter diagram showing differentially expressed proteins in the MHCC-97H and MHCC-97L cell lines (p<0.05). (F) The total intensity of crotonylation was higher in MHCC-97H cell line (p<0.05). Figure S2. Crotonylome in differential invasive HCC cell lines (A-B) KEGG analysis: (A) Reactome analysis (B) differential crotonylated proteins. (C) Heatmap of known metastatic-related proteins in MHCC-97H and MHCC-97L cell lines. Figure S3. WB analysis of total crotonylation of HCC cell lines after NaCr treatment. Figure S4. Crotonylation was positively correlated with HCC cell migration and invasion (A)Cell Morphological changes in SMMC7721 cell line after 25mM of NaCr treatment. (B) Migration and Invasion assays showed SMMC7721 cells had higher invasive potential after 25mM of NaCr treatment. Data presented as mean ± SD. *p < 0.01, **p<0.001. (Student’s t test). (C) Wound heal assays showed SMMC7721 cells had greater migration capacity after 25mM of NaCr treatment. Data presented as mean ± SD. *p < 0.01. (Student’s t test). (D) WB analysis of the changes in the expression of EMT-related proteins after 25mM of NaCr treatment. Figure S5. Lysine 74 crotonylation of SEPT2 was identified (A)Scatter diagram based on the false discovery rate (FDR) of protein expression and crotonylation. (B) SEPT2 K318 is less evolutionarily conserved in seven species compared with K74. K318 of SEPT2 was highlighted in red. (C) Coomassie blue of purified Flag-tagged SEPT2. (D) Dot blotting assay of site-specific antibody of SEPT2-K74 crotonylation (K74Cr). (E)WB analysis of overexpression of Flag-t [file 13578_2023_996_MOESM1_ESM.docx]

Additional file for

**SEPT2 Crotonylation Promotes Metastasis and Recurrence in hepatocellular carcinoma and is Associated with Poor Survival**

Xin-yue Zhang*，Ze-xian Liu*，Yi-fan Zhang*，Li-xia Xu，Meng-ke Chen，Yu-feng Zhou，Jun Yu，Xiao-xing Li^#^，Ning Zhang^#^

#Corresponding author. Xiao-xing Li, Email: [lixiaox23@mail.sysu.edu.cn](mailto:lixiaox23@mail.sysu.edu.cn);

Ning Zhang, Email: [zhangn5@mail.sysu.edu.cn](mailto:zhangn5@mail.sysu.edu.cn).

**This PDF file includes:**

Figs. S1 to S12

Tables S1 to S3

Supplementary Methods

Data S1

Figure S1.


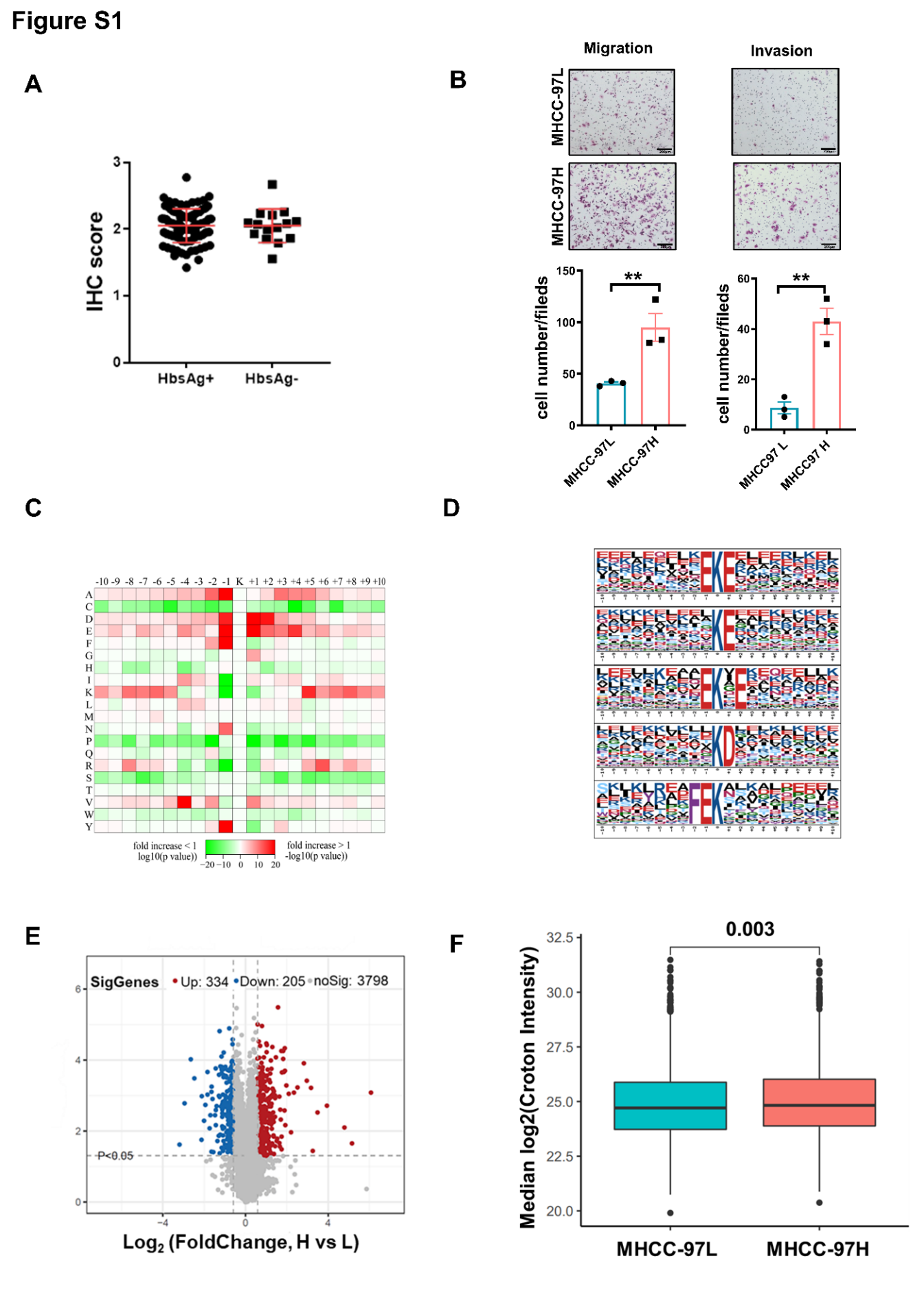


**Figure S1.** **Crotonylome in HCC cells**

(A)IHC analysis showed the crotonylation level are similar in HBV positive and negative HCC. (B) Migration and invasion assays showing that MHCC-97H cells had higher invasive potential than MHCC-97L cells. The data are presented as the means ± SD. *p < 0.05, **p < 0.01 (Student’s t test). (C-D) Motif analysis of all identified crotonylated sites. (E)Scatter diagram showing differentially expressed proteins in the MHCC-97H and MHCC-97L cell lines (p<0.05). (F) The total intensity of crotonylation was higher in MHCC-97H cell line (p<0.05).

Figure S2.


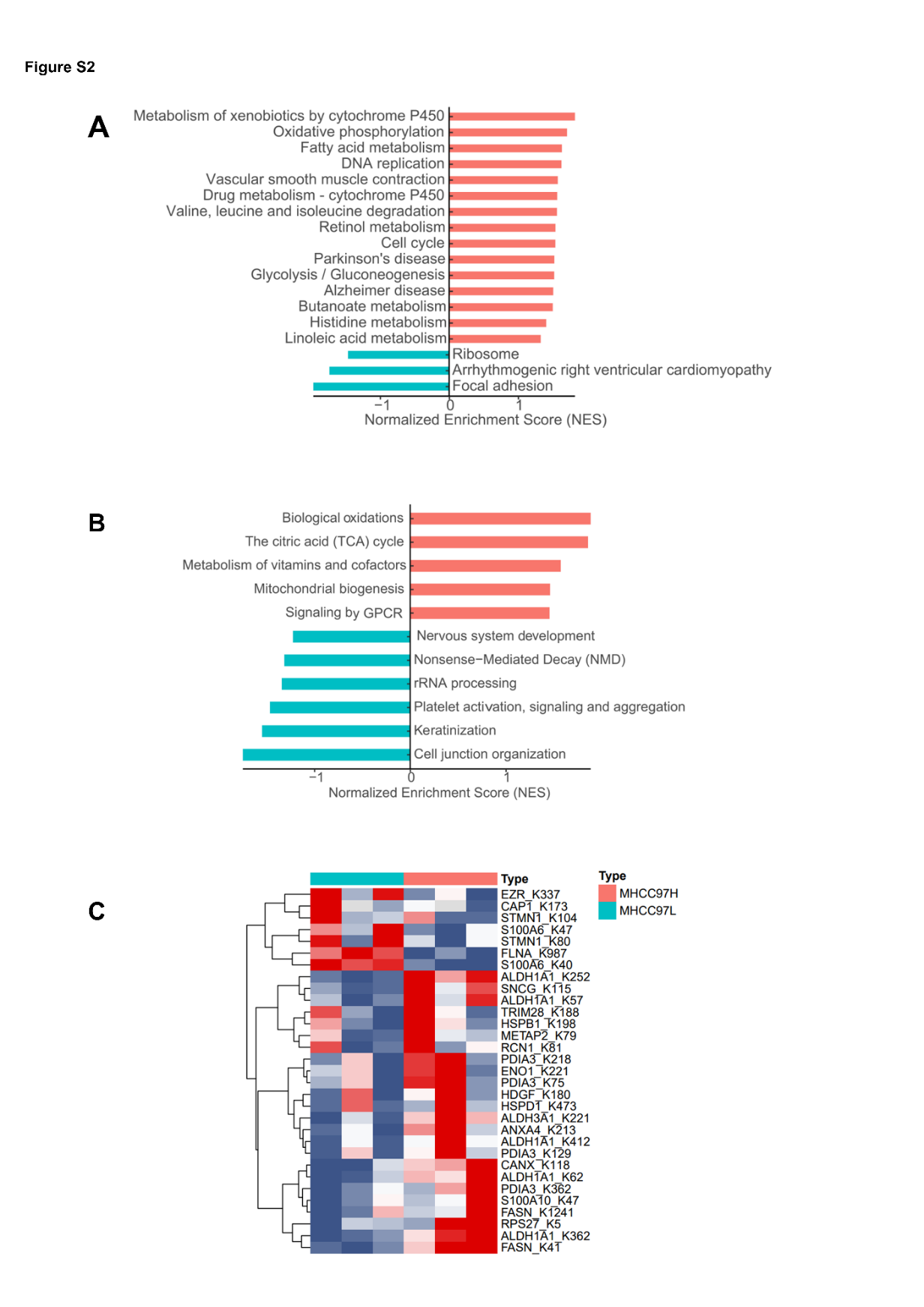


**Figure S2. Crotonylome in differential invasive HCC cell lines**

(A-B) KEGG analysis: (A) Reactome analysis (B) differential crotonylated proteins.

(C) Heatmap of known metastatic-related proteins in MHCC-97H and MHCC-97L cell lines.

Figure S3.


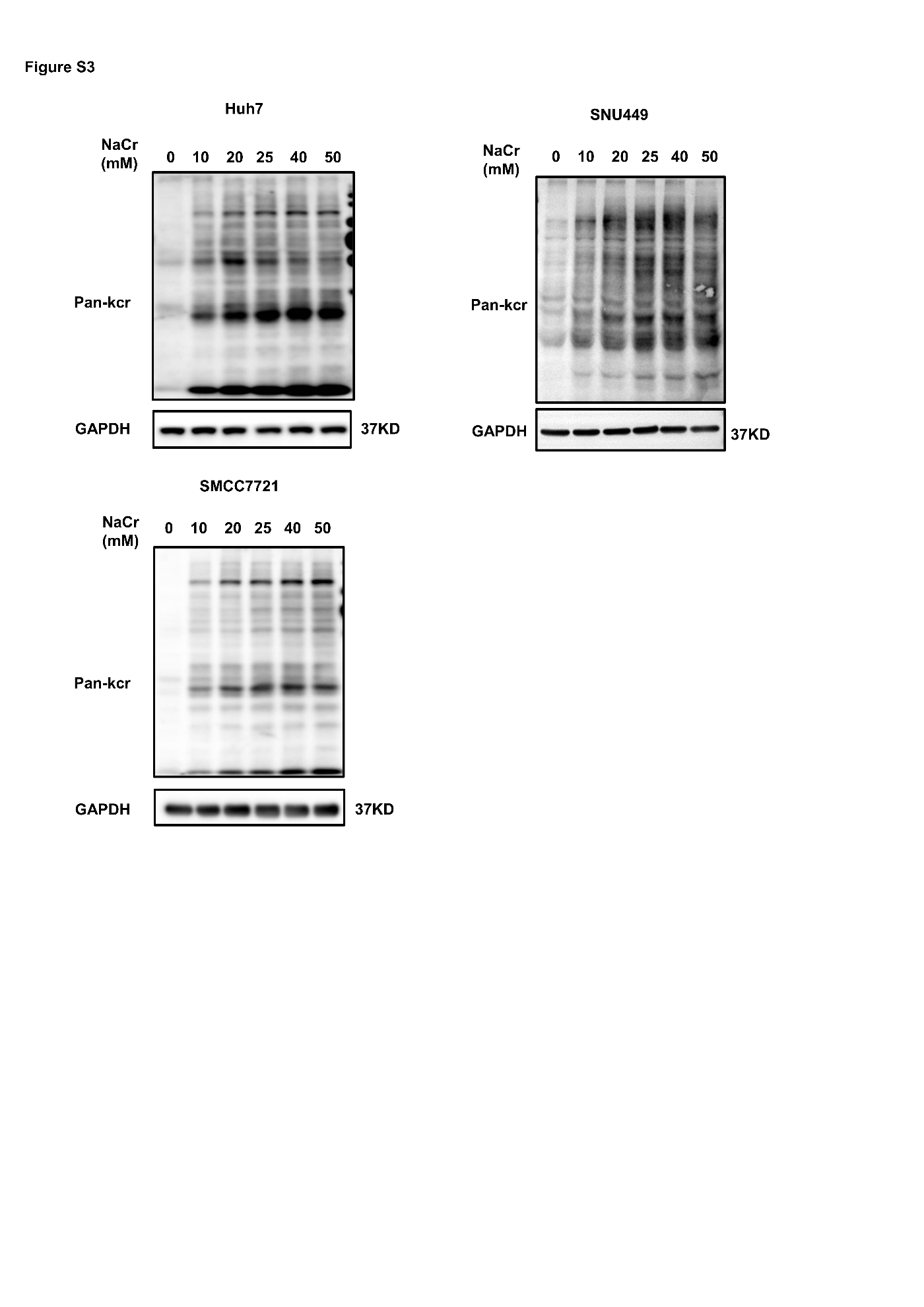


**Figure S3.** **WB analysis of total crotonylation of HCC cell lines after NaCr treatment.**

Figure S4.


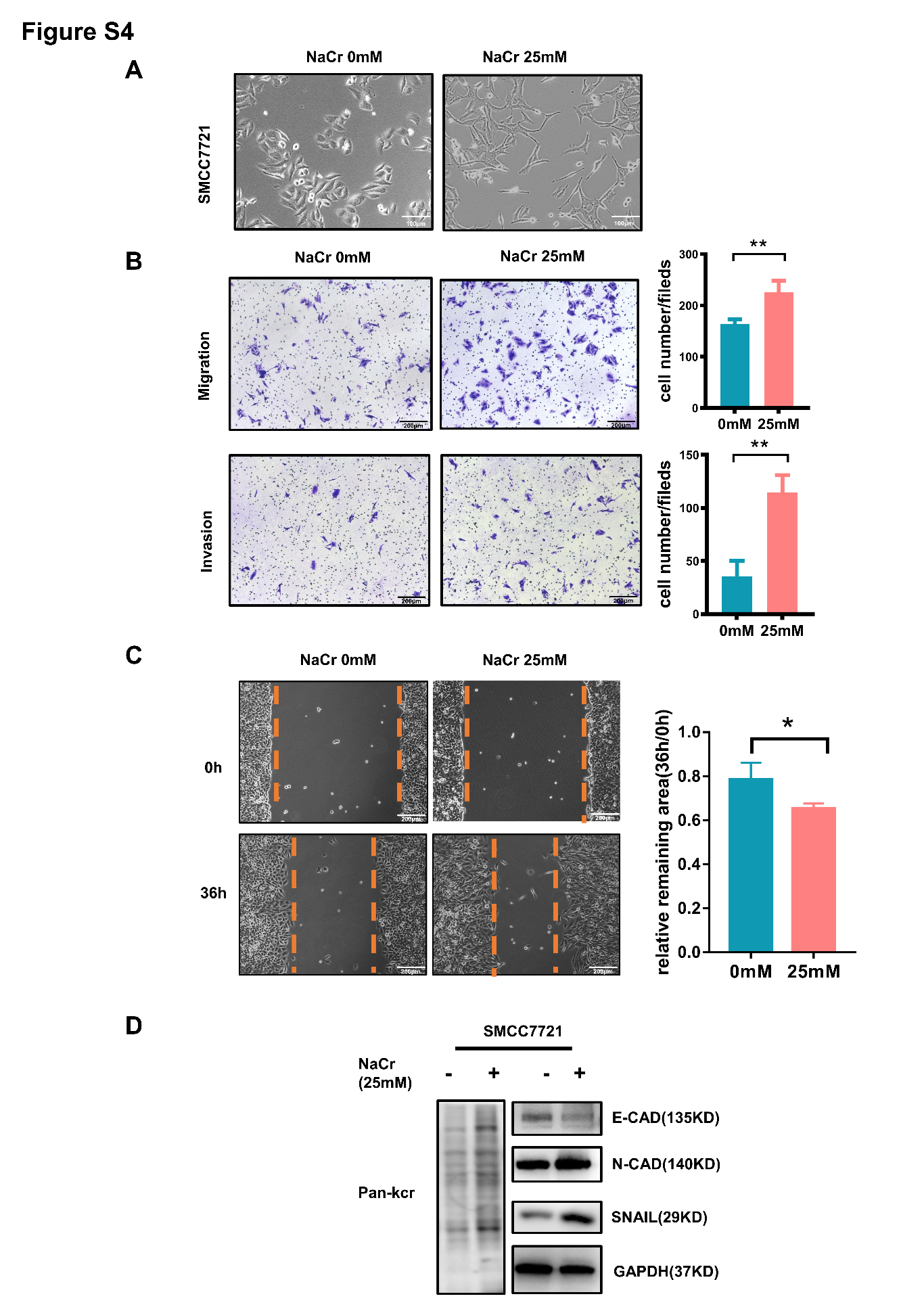


**Figure S4. Crotonylation was positively correlated with HCC cell migration and invasion**

(A)Cell Morphological changes in SMMC7721 cell line after 25mM of NaCr treatment. (B) Migration and Invasion assays showed SMMC7721 cells had higher invasive potential after 25mM of NaCr treatment. Data presented as mean ± SD. *p < 0.01, **p<0.001. (Student’s t test). (C) Wound heal assays showed SMMC7721 cells had greater migration capacity after 25mM of NaCr treatment. Data presented as mean ± SD. *p < 0.01. (Student’s t test). (D) WB analysis of the changes in the expression of EMT-related proteins after 25mM of NaCr treatment.

Figure S5.


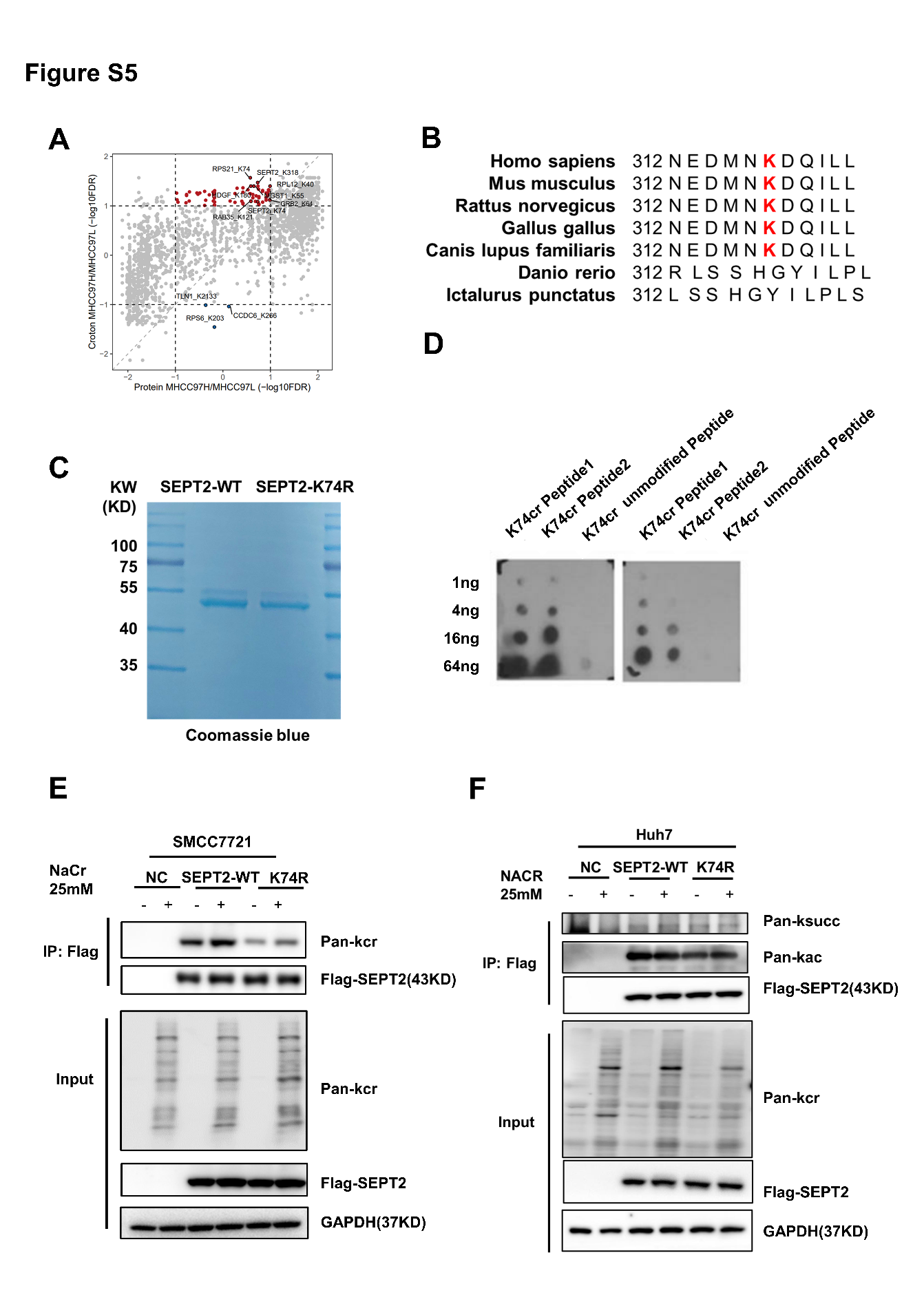


**Figure S5. Lysine 74 crotonylation of SEPT2 was identified**

(A)Scatter diagram based on the false discovery rate (FDR) of protein expression and crotonylation. (B) SEPT2 K318 is less evolutionarily conserved in seven species compared with K74. K318 of SEPT2 was highlighted in red. (C) Coomassie blue of purified Flag-tagged SEPT2. (D) Dot blotting assay of site-specific antibody of SEPT2-K74 crotonylation (K74Cr). (E)WB analysis of overexpression of Flag-tagged SEPT2-WT and K74R, pan-crotonylation in cells and crotonylation on SEPT2 with or without NaCr treatment. (F) WB analysis of SEPT2 acetylation (Kac) and succinylation (Ksucc) in cells with or without NaCr treatment.

Figure S6.


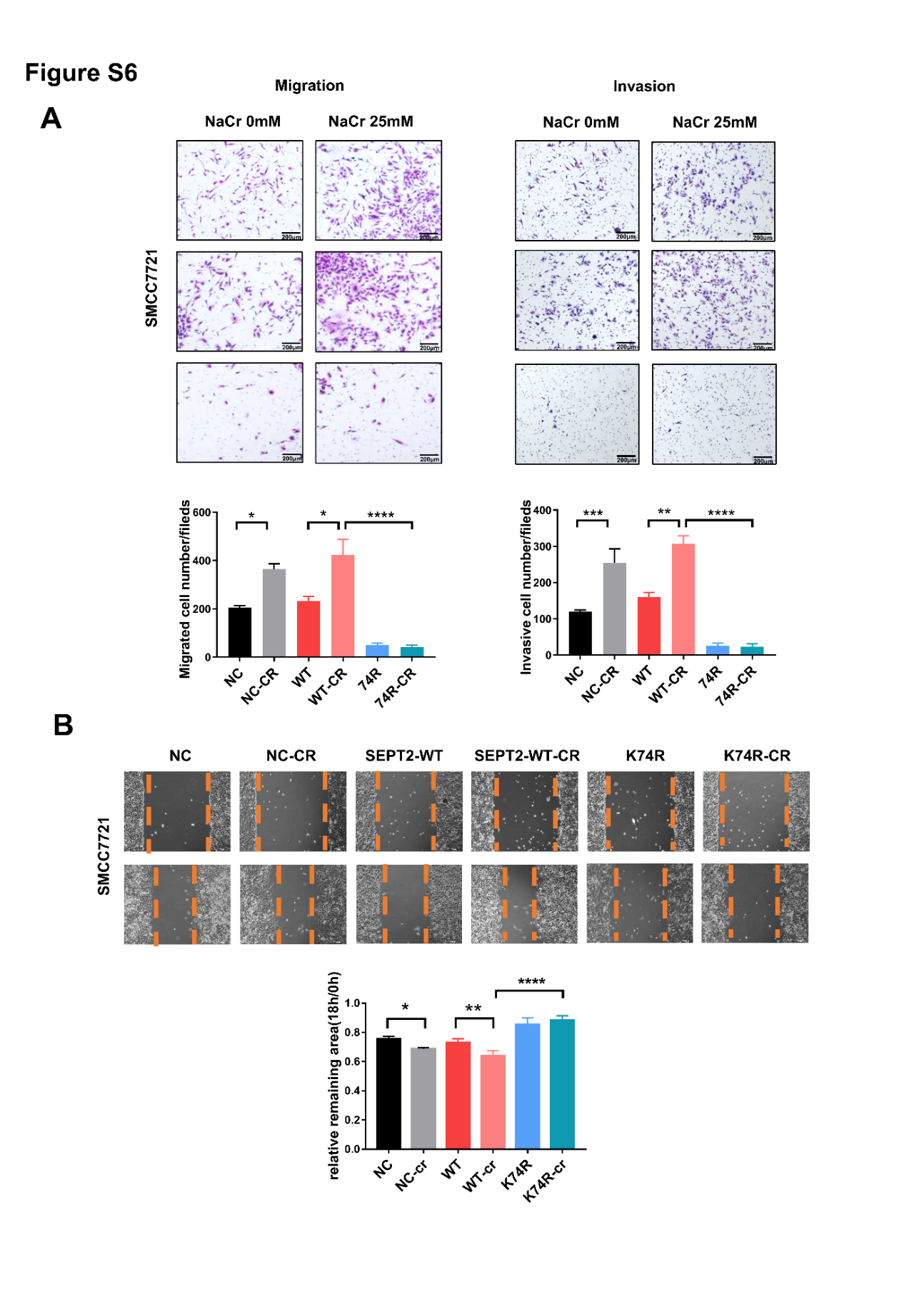


**Figure S6. SEPT2-K74R Inhibited Cell Migration and Invasion in vitro**

(A-B) Overexpression of SEPT2-K74R inhibited the ability of cell migration and invasion in SMMC7721 cells with and without 25mM of NaCr treatment.

Data presented as mean ± SD. *p < 0.05, **p < 0.01, ***p < 0.001, ****p<0.0001 (One-way ANOVA).

Figure S7.


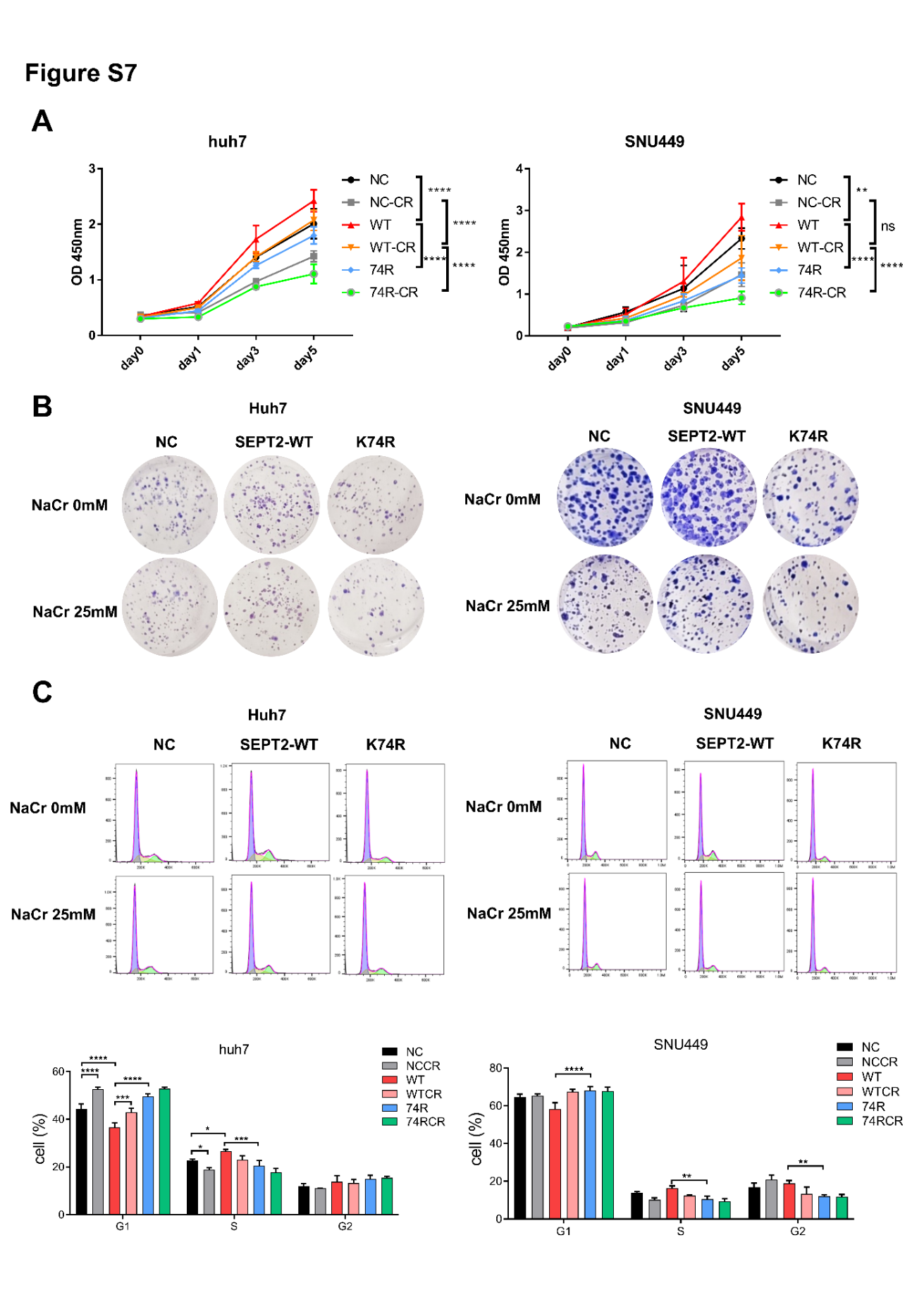


**Figure S7. SEPT2-K74R and NaCr Inhibited Cell Oroliferation and Cell Cycle.**

CCK8 analysis (A), colony formation assay (B) and cell cycle assay (C) of SEPT2-WT and SEPT2-K74R overexpression cells with and without NaCr treatment.

Figure S8.


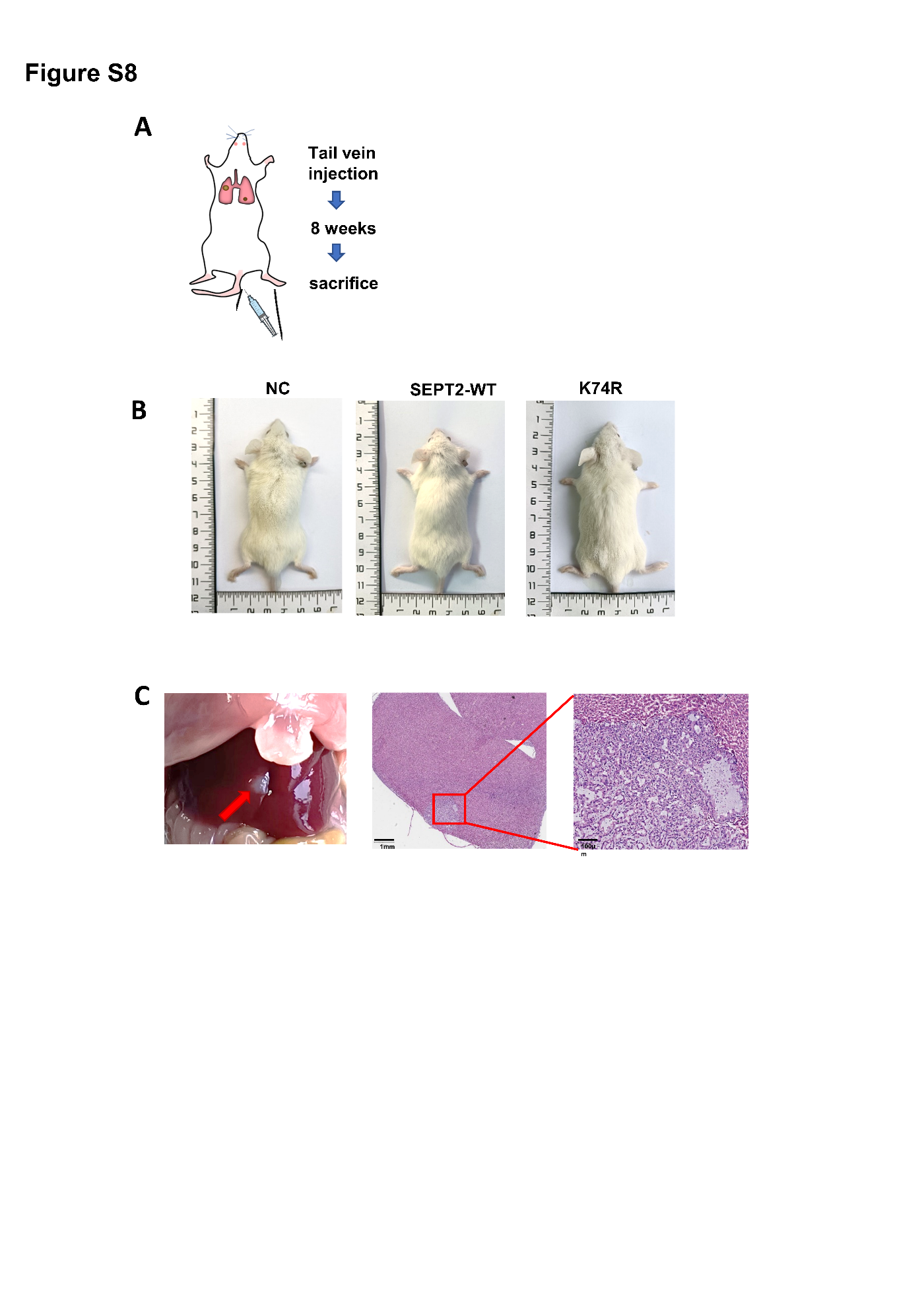


**Figure S8. SEPT2-K74R Inhibited Cell Migration and Invasion in tail vein injection mode**

(A)Flowchart of the tail vein injection model. Huh7 stable cells were injected into the tail vein of NCG mice (8 mice each group). Mice were sacrificed 8 weeks after injection. (B) Mice in SEPT2-K74R group had better nutrition. (C) Representative images of liver metastases in tail vein injection model.

Figure S9.


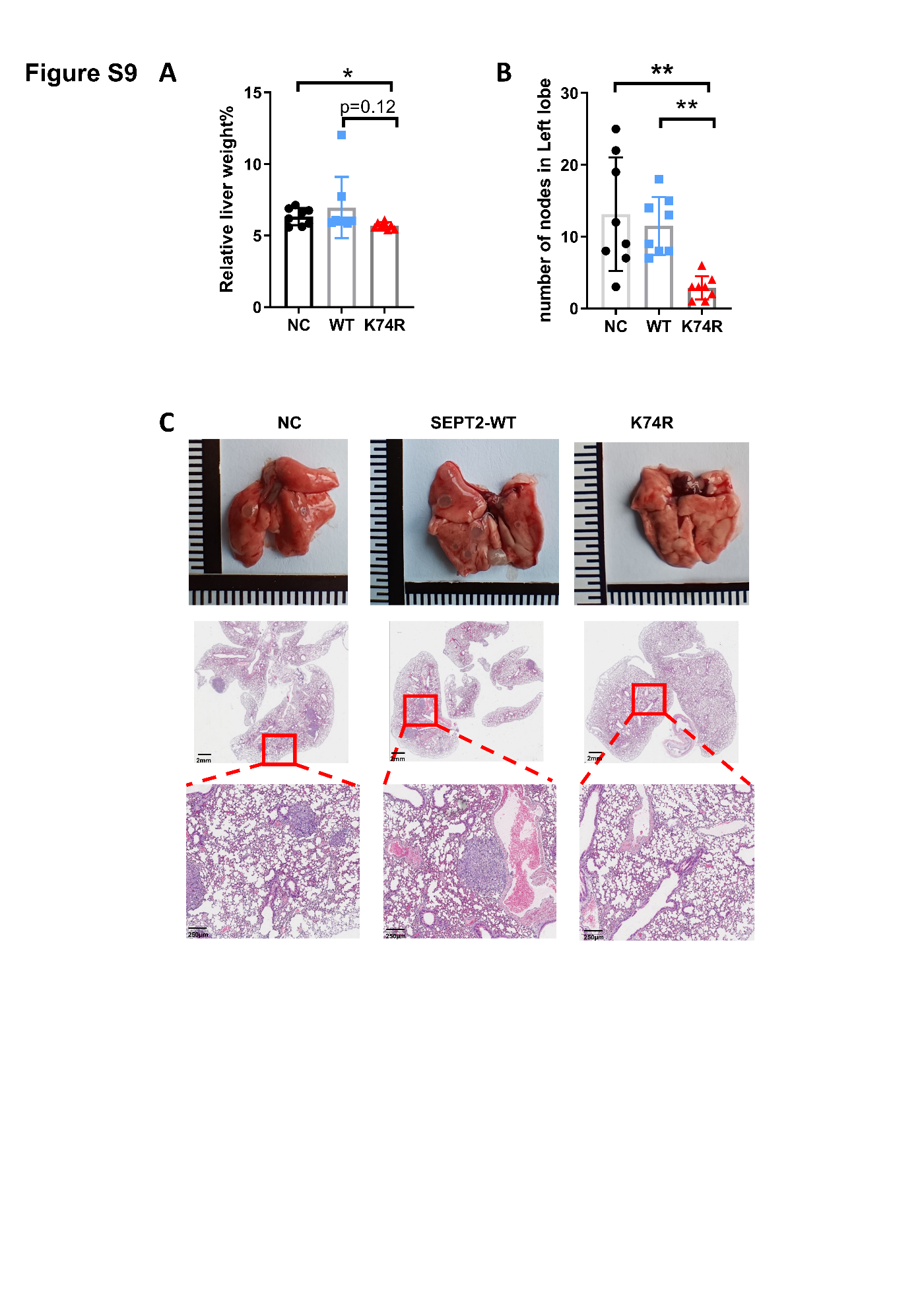


**Figure S9. SEPT2-K74R Inhibited Cell Migration and Invasion in orthotopic liver tumor implantation mouse model**

(A)Quantitative comparison of tumor burden by calculating liver weight to body weight ratio of each mouse in orthotopic liver tumor implantation mouse model. (B) Quantitative comparison of the number of liver metastases in left lobe. Mice in the SEPT2-K74R group had fewer liver metastasis in orthotopic liver tumor implantation mouse model. (C) Representative images of lung metastases in orthotopic liver tumor implantation mouse model. Data presented as mean ± SD. *p < 0.05, **p < 0.01, ***p < 0.001, ****p<0.0001 (One-way ANOVA).

Figure S10.


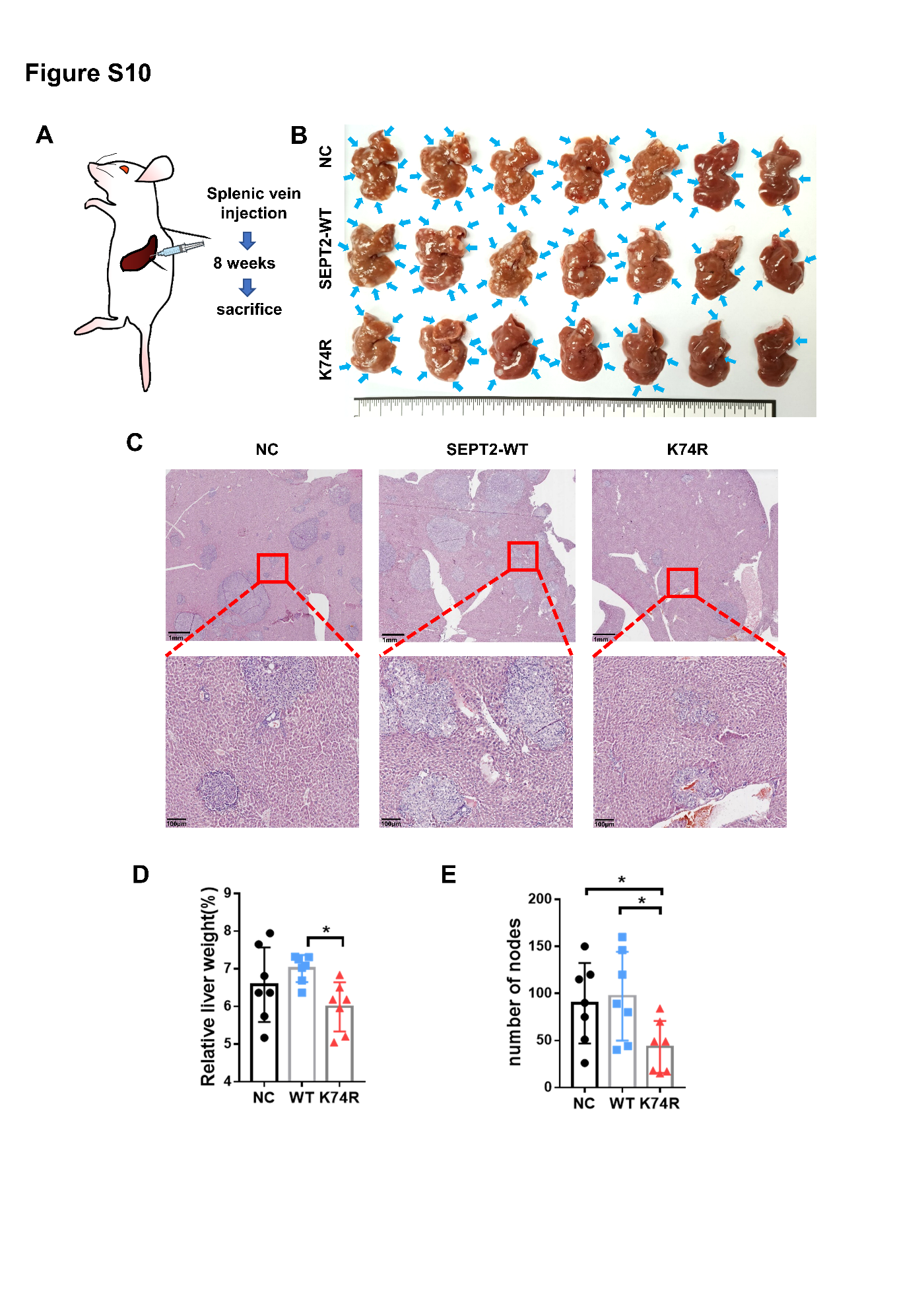


**Figure S10.** **SEPT2-K74R Inhibited Cell Migration and Invasion in splenic vein injection mouse model**

(A)Flowchart of the splenic vein injection mouse model. Huh7 stable cells were injected into the tail vein of NCG mice (7 mice each group). Mice were sacrificed 8 weeks after injection. (B) Representative images of liver metastases in the splenic vein injection mouse model. Blue arrows showed the liver metastases. (C) Representative images of liver metastases in the splenic vein injection mouse model. Tissues were stained by HE. (D) Quantitative comparison of tumor burden by calculating liver weight to bod weight ratio of each mouse in the splenic vein injection mouse model. (E) Quantitative comparison of the number of liver metastases in each group. Mice in the SEPT2-K74R group had fewer liver metastasis. Data presented as mean ± SD. *p < 0.05 (One-way ANOVA).

Figure S11.


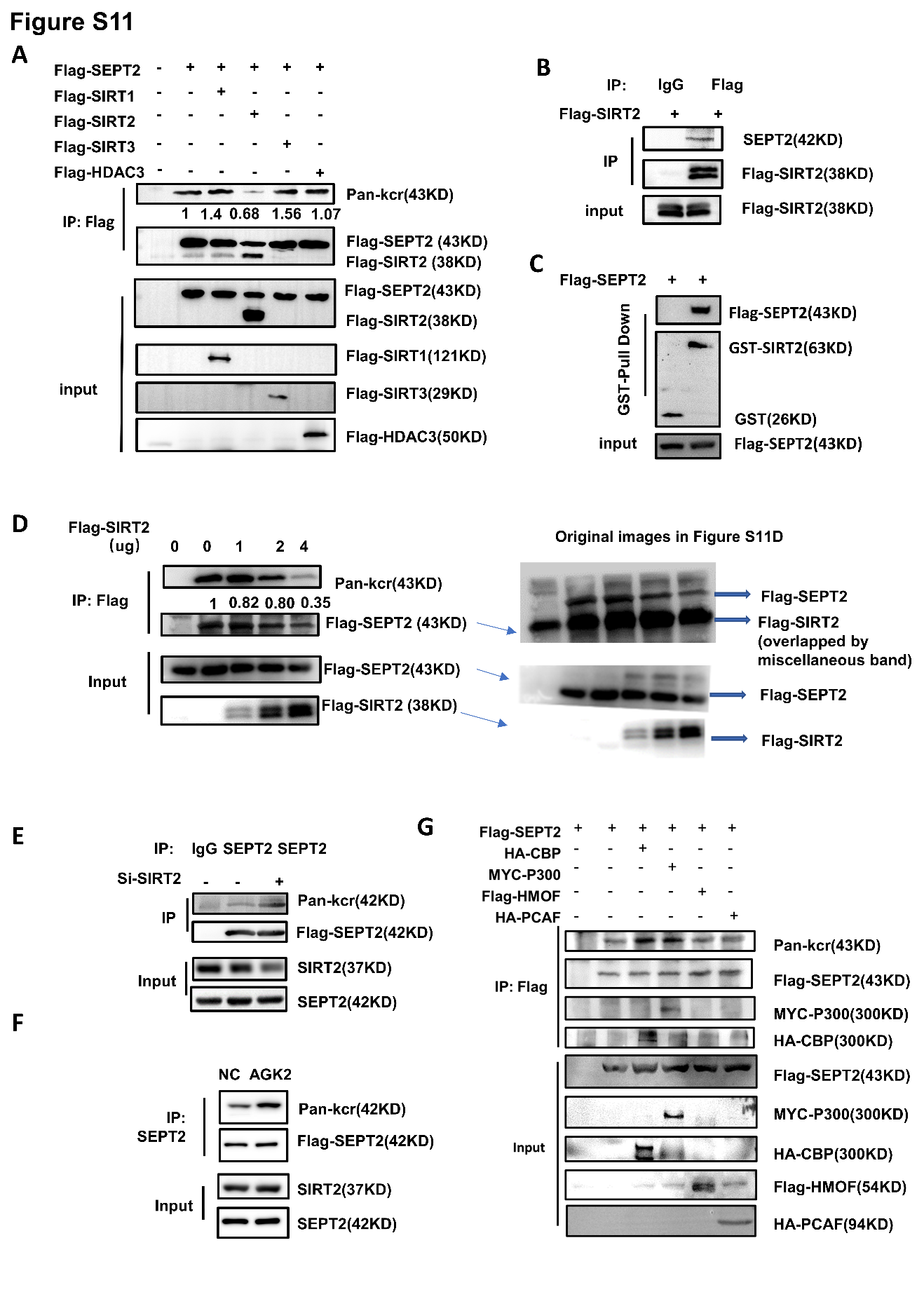


Figure S11. SIRT2 decrotonylated SEPT2.

(A)SIRT2 decreased crotonylation of SEPT2. Whole-cell lysates were immunoprecipitated with anti-Flag antibody, and precipitated proteins were detected by anti-Flag and anti-pan-kcr antibodies. (B) SIRT2 interacts with SEPT2. Whole-cell lysates were immunoprecipitated with control IgG and anti-Flag antibody, and precipitated proteins were detected by anti-SEPT2 and anti-Flag-SIRT2 antibodies. (C) SIRT2 interacts with SEPT2 in vitro. Purified GST-tagged SIRT2 protein were immunoprecipitated with Purified Flag-tagged SEPT2 and GST antibody-conjugated beads. (D-E) WB analysis of crotonylation of SEPT2. SEPT2 crotonylation was detected with different amount of SIRT2 (D) and SIRT2 interference (E). (F) WB analysis of crotonylation of SEPT2 after AGK2 treatment. (G)CBP and P300 interact with SEPT2 and could crotonylate SEPT2.

Figure S12.


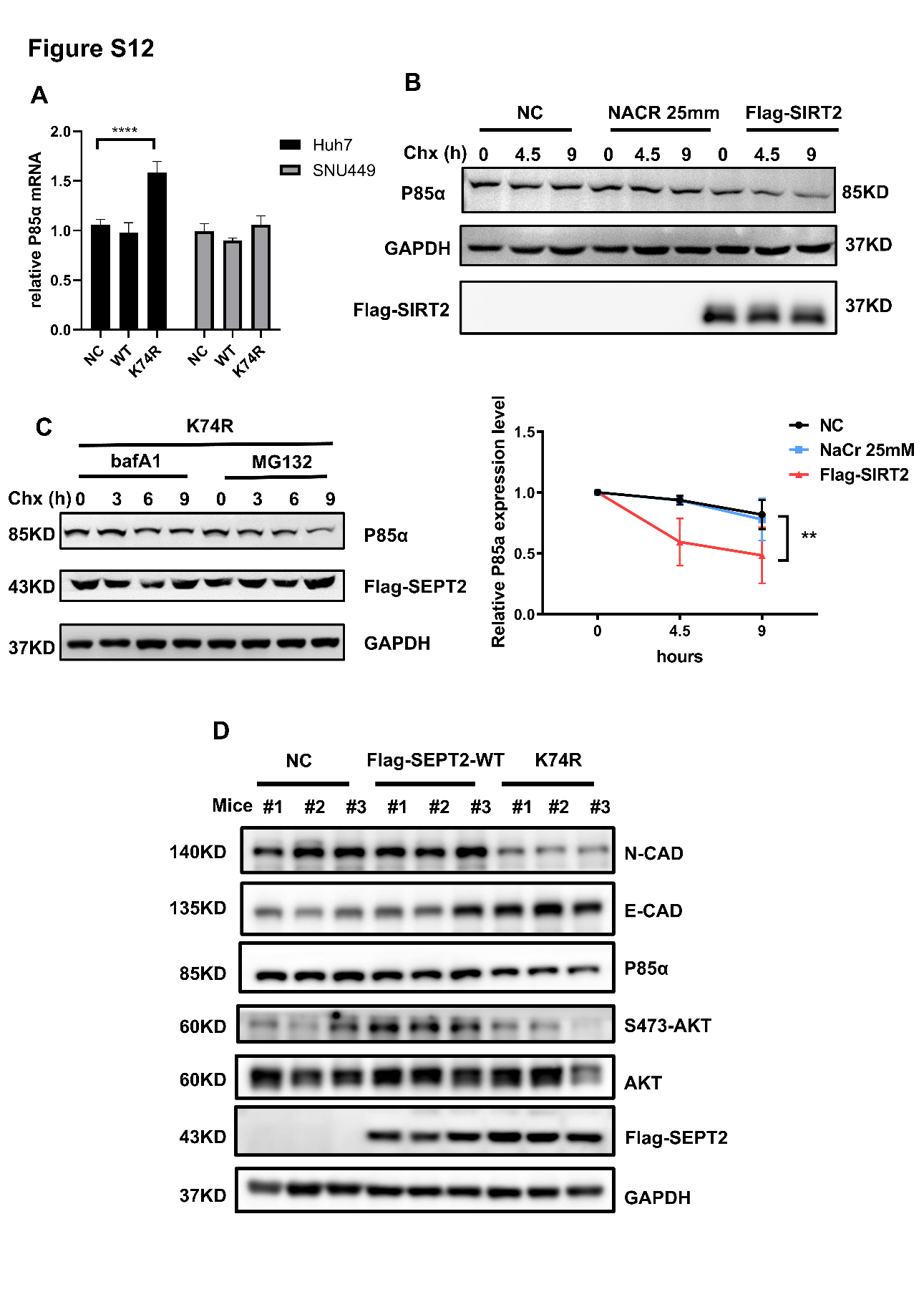


**Figure S12. SEPT2-K74R downregulated P85α**

(A)qRT-PCR analysis of relative fold change of P85α mRNA expression in NC, SEPT2-WT (WT) and SEPT2-K74R (K74R) groups. Data presented as mean ± SD. *p < 0.05, **p < 0.01, ***p < 0.001, ****p<0.0001 (One-way ANOVA). (B) SIRT2 overexpression decreased P85α stability. SNU449 cells were treated with CHX and P85α protein was determined by WB (up panel). The down panel showed relative protein level of different groups. Error bars represent ±SD of triplicate experiments. T. *p < 0.05, **p < 0.01, ***p < 0.001, ****p<0.0001 (One-way ANOVA). NC, negative control. (C) BafA1 increased P85α stability under SEPT2-K74R overexpression. (D) WB analysis of the expression levels of NCAD, ECAD, P85α, Phospho-AKT （S473-AKT）, AKT in mouse tumor tissues.

Figure S13.


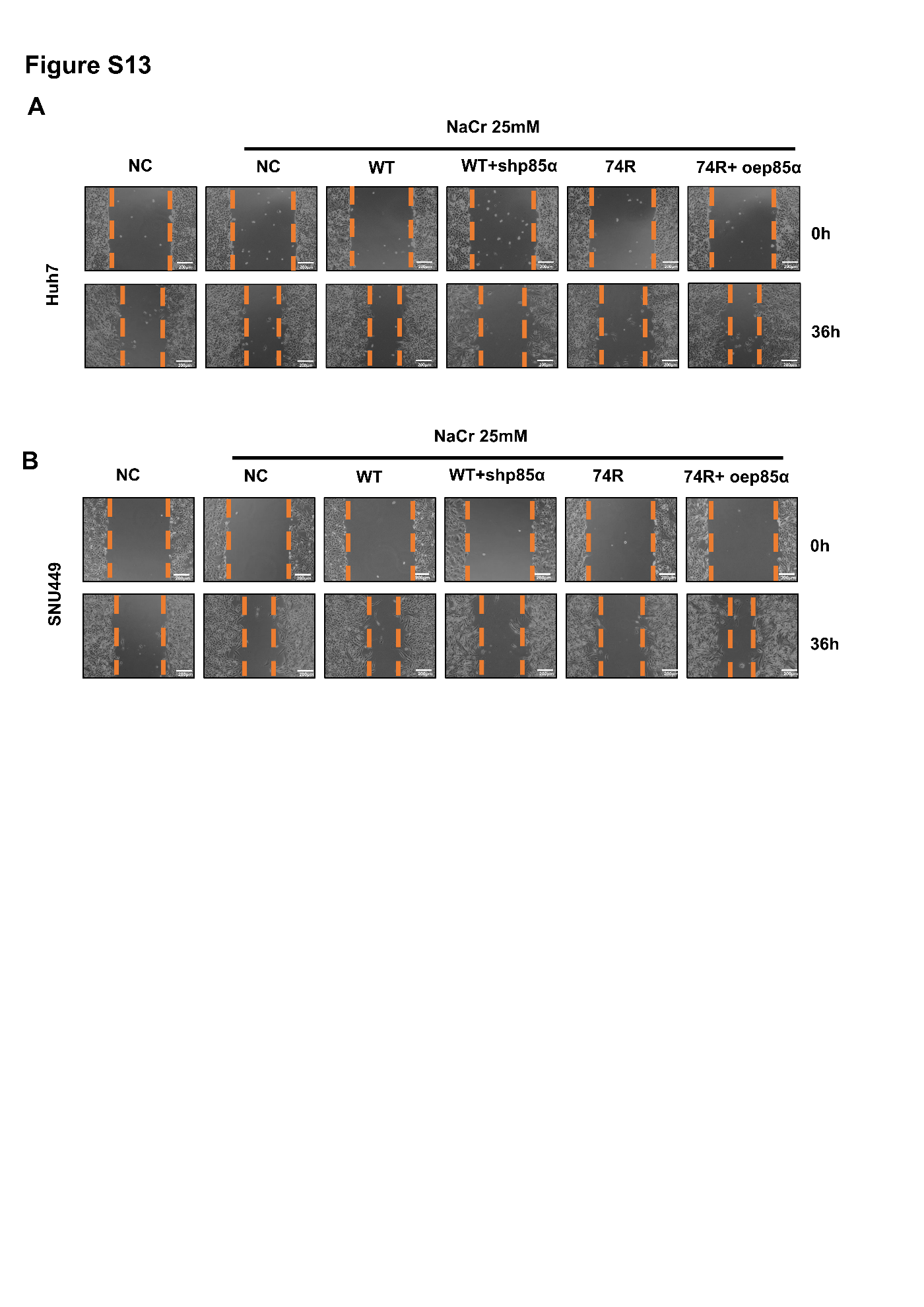


**Figure S13. Crotonylation facilitated cell invasive capability in SEPT2-K74Cr -P85α-Akt pathway**

(A-B) P85α rescued the function of SEPT2 under NaCr treatment. Knock down of P85α inhibited cell migration under NaCr treatment; Overexpression of P85α in SEPT2-K74R rescued the ability of cell migration in Huh7 (A) and SNU449 (B) cells.

Figure S14.


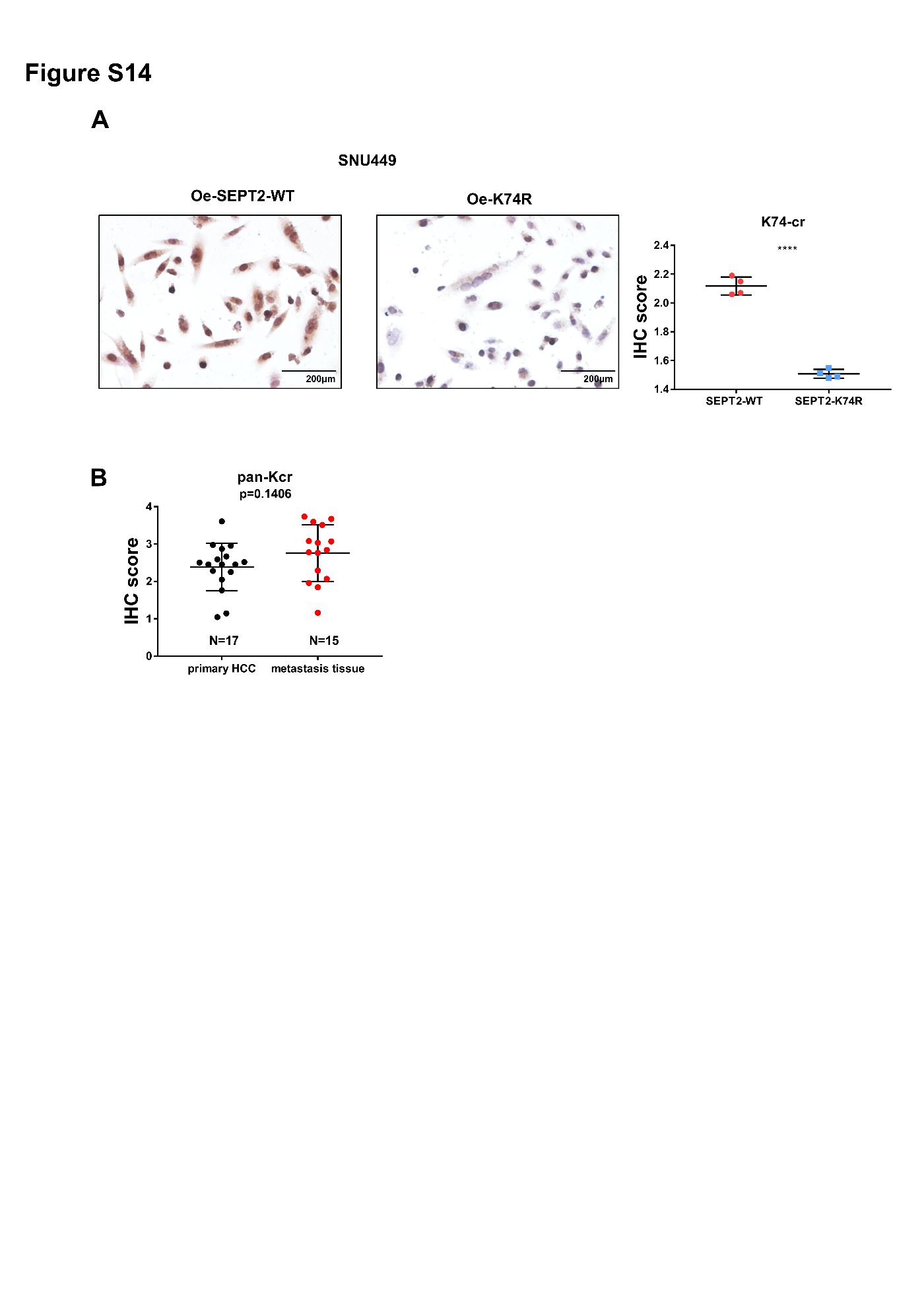


**Figure S14.**

1. IHC assays in adherent SNU449 cells. We used IHC staining to examine the K74-cr level in stably overexpressed SEPT2 WT and SEPT2-K74R adherent HCC cells, as positive and negative control respectively.
2. Quantitative comparison of the Pan-Kcr IHC scores in metastasis tissue and primary HCCs in Tissue microarray (HLivH060CD03)

Table S1.

| Table S1. Distribution of Patients characteristics by survival status | | | | | | |
| --- | --- | --- | --- | --- | --- | --- |
|  | | Alive | | Dead | | P value |
|  |  | n | % | n | % |  |
| BCLC stage | Early stage | 64 | 67.4 | 31 | 32.6 | 0.005 |
|  | Late stage | 12 | 38.7 | 19 | 61.3 |  |
|  |  |  |  |  |  |  |
| Mean age, y±SD | | 50±2.5 | | 54±3.2 | | 0.07 |
|  | |  | |  | |  |
| gender | MALE | 65 | 59.1 | 45 | 40.9 | 0.461 |
|  | FEMALE | 11 | 68.8 | 5 | 31.3 |  |
|  |  |  |  |  |  |  |
| SEPT2-K74cr level | Low expression | 37 | 71.2 | 15 | 28.8 | 0.037 |
|  | High expression | 39 | 52.7 | 35 | 47.3 |  |

Table S2.

| Table S2. Distribution of Patients characteristics by recurrence status | | | | | | |
| --- | --- | --- | --- | --- | --- | --- |
|  | | Health | | Recurrence | | P value |
|  |  | n | % | n | % |  |
| BCLC stage | Early stage | 48 | 52.2 | 44 | 47.8 | 0.217 |
|  | Late stage | 10 | 38.5 | 16 | 61.5 |  |
|  |  |  |  |  |  |  |
| Mean age, y±SD | | 50.5±3.3 | | 52.9±2.5 | | 0.07 |
|  | |  | |  | |  |
| gender | MALE | 50 | 48.5 | 53 | 51.5 | 0.729 |
|  | FEMALE | 8 | 53.3 | 7 | 46.7 |  |
|  |  |  |  |  |  |  |
| SEPT2-K74cr level | Low expression | 27 | 60.0 | 18 | 40.0 | 0.064 |
|  | High expression | 31 | 42.5 | 42 | 57.5 |  |

Table S3.

| Table S3. Univariate Cox Regression Analysis of Potential Recurrence Factors for HCC Patients | | | |
| --- | --- | --- | --- |
| Variable | | RR(95% confidence interval) | P value |
| BCLC stage | Low stage | 0.647(0.365-1.148) | 0.137 |
|  | High stage | 1.00 |  |
| Age | | 1.010(0.989-1.032) | 0.364 |
| gender | MALE | 0.996(0.439-2.125) | 0.931 |
|  | FEMALE | 1.00 |  |
| SEPT2-K74cr level | Low expression | 0.575(0.330-0.999) | 0.050 |
|  | High expression | 1.00 |  |

Supplementary Methods

**Cell cultures**

The MHCC97L, MHCC97H, Huh7 and HEK293T human cell lines were grown in DMEM (GIBCO, Grand Island, USA). SNU449 and SMMC7721 cell lines were grown in RPMI 1640 medium (GIBCO). All media were supplemented with 10% fetal bovine serum (FBS, GIBCO). The HEK293F cell line was grown in Union 293 medium (Union-biotech, Shanghai, China). All cell lines were purchased from BLUEFBIO (Shanghai, China) and Cellcook Biotech Co. Ltd (Guangzhou, China). The cell lines were free from mycoplasma infection and authenticated by short tandem repeat (STR) assay.

**RNA isolation, reverse transcription and qRT–PCR**

Total RNA was extracted by TRIzol reagent (Invitrogen, New York, USA) following the manufacturer’s instructions. Reverse transcription was performed with the PrimeScript RT reagent kit (TaKaRa, Japan). The cDNA samples were diluted 1:5 for gene expression analysis by qRT–PCR and gene amplification by PCR. qRT–PCR was carried out with TB Green^TM^ Premix Ex TaqTM II (TaKaRa) in a LightCycler 480 real-time PCR system (Roche, Basle, Switzerland). Each sample was analyzed three times, and relative mRNA expression was calculated using GAPDH as the internal control.

**Plasmids and lentivirus infection**

Flag-tagged full-length human wild-type (WT) SEPT2, K74R and K318R SEPT2 mutants and full-length SIRT2 were cloned into a retroviral (pCDH), PcDNA 3.1+ and pGEX-4t-1 vector. SIRT1, SIRT3, HDAC3, CBP, P300, HMOF and PCAF were cloned into a PcDNA 3.1+ vector. Full-length human PIK3R1 (P85α) was cloned into a pCDH vector. Short interfering RNAS (shRNAs) against PIK3R1 were purchased from GENE, China. Transient plasmid transfection was performed using Lipofectamine 3000 according to the manufacturer’s instructions (Invitrogen) and DNA transfection reagent (NEOFECT, Beijing, China). The medium was changed 24 hours after transfection, and the cells were collected 48 hours after transfection.

To produce lentivirus, HEK293T cells were seeded into 6 cm cell culture plates before transfection. Then, 15 µl of P3000 was diluted in 250 µl of Opti-MEM (GIBCO) with 6 µg of pCDH plasmid, 4 µg of psPAX2 and 2 µg of pMD2.G, and then added to 250 µl of Opti-MEM (with 15 µl of Lipo3000), which was added to the culture plate wells. After 48 hours, the cell supernatant was collected and filtered with a 0.45 µm low protein-binding membrane (Millipore Steriflip). Cells grown to 80% confluence were transfected with the lentivirus in the presence of 8 μg/ml polybrene (Solarbio, Beijing, China). Puromycin (Solarbio) was used to select stably transfected cells.

siSIRT2: GGACGAGCUGACCUUGGAATT/UUCCAAGGUCAGCUCGUCCTT

**Western blot analysis and immunoprecipitation (IP)**

Total protein was extracted with RIPA lysis buffer (CST, Boston, USA) containing protease inhibitors and phosphatase inhibitors (Sigma, Darmstadt, Germany). The protein concentration was measured with a Pierce^TM^ BCA Protein Assay Kit (Thermo). Protein samples (15 µg) were separated on a Bis/Tris-polyacrylamide gel (Epizyme, Shanghai, China) by electrophoresis and then blotted onto PVDF membranes (Merck Millipore, Germany). The blots were incubated with the primary antibodies overnight at 4°C and with secondary antibodies (CST) for 1 hour at room temperature. An Omni-ECL™ Femto Light chemiluminescence Kit (Epizyme) was used to visualize the proteins. For IP, IP lysis buffer (150 mM NaCl, 50 mM Tris-HCl, 0.5% Nonidet P40 [CWBIO], 5 mM EDTA, proteinase inhibitor cocktail [CWBIO, China] and PMSF [CWBIO], pH=7.4) was used to extract total protein. The cell lysate was incubated with appropriate antibodies and protein-A/G beads (Beyotime) for 12 hours. The immunoprecipitates were washed five times with cold IP lysis buffer and eluted with SDS loading buffer after boiling for 10 min subjected to WB.

**Antibodies and regents**

Anti-SEPT2 (1:1,000 for western blotting (WB), no. 179436), anti-GRB2 (1:1,000 for WB, no. 32037), and anti-P85α (1:1000 for WB, no. 191606) antibodies were purchased from Abcam (Cambridge, UK). Anti-RAB35 (1:1000 for WB, no. 9690S), Anti-E-Cadherin (1:2000 for WB and immunohistochemistry [IHC], no. 20874-1) and anti-GST (1:1000 for WB, no. 66001-2) antibodies were purchased from Proteintech (Chicago, USA). Anti-N-Cadherin and Anti-GAPDH were purchased from CST. Anti-pan-crotonylation (pan-Kcr; 1:1000 for WB) and anti-crotonylated SEPT2 at K74 (SEPT-74Kcr; 1:1000 for WB and IHC) antibodies were purchased from PTM BioLab (Hangzhou, China). Anti-FLAG-tagged antibody (1:3,000 for WB) was purchased from Abmart (Shanghai, China). Bafilomycin A1 (BafA1), cycloheximide (CHX), and MG132 were purchased from MCE (New Jersey, USA). Crotonic acid was purchased from Macklin (Shanghai, China). AGK2 was purchased from beyotime (Shanghai, China).

***In vitro* migration and invasion assays**

A 24-well plate containing chamber inserts with 8 mm pores (Corning, New York, USA) was utilized to evaluate the migration and invasion of HCC cells. A total of 1×10^5^ cells were positioned in the upper chamber precoated with 10% Matrigel (BD Biosciences, New York, USA) (invasion assay) or without with Matrigel (migration assay). In each lower chamber, 750 μL of culture medium supplemented with 10% FBS was added. Cells were incubated at 37 °C and allowed to migrate for 8 hours or invade for 24 hours. After incubation, the cells that had migrated or invaded through the pores were fixed with 4% paraformaldehyde and stained with 0.1% crystal violet. Then, the cells were photographed and counted.

**Wound-healing assay**

HCC cells were grown until they reached confluence, then scratching the cells using a sterile 200µl pipette tip. Cells were washed once with PBS to remove floating cells and then changed to medium containing 1% FBS. The closure of the wound was monitored and captured by a microscope every 12 hours.

**Cell viability assay**

The cell viability assay was performed by Cell Counting Kit-8 (CCK8, Dojindo, Shanghai, China) according to the manufacturer’s instructions. Briefly, 1×10^3^ cells per well were seeded into a 96-well plate. 90 µl of culture medium and 10 µl of CCK8 were mixed and were then added into the cells at 0-day, 1-day, 3-day and 5-day after seeding. The mixture was incubated at 37°C for 2 hours, and the optical density (OD) was measured at 450 nm wavelength.

**Colony formation assay**

1×10^3^of cells were seeded into a 6-well plate containing complete medium with or without NaCr treatment. After 2 weeks’ incubation, the formatted colony were fixed with 4% paraformaldehyde and stained with 0.1% crystal violet.

**Cell cycle assay**

1×10^5^of cells were seeded into a 6-well plate containing complete medium with or without NaCr treatment. After 24 hours’ incubation, cell cycle was detected by PI/RNase Staining Kit (Dojindo, Shanghai, China) followed with flow cytometry analysis with CytoFLEX (Beckman Coulter, USA) and Flowjo software of 8.1 version.

**H&E and IHC staining**

For H&E staining, paraffin sections were dewaxed in xylene, rehydrated using sequentially decreasing concentrations of ethanol and subsequently washed in PBS and stained with H&E. After staining, the sections were dehydrated in increasing concentrations of ethanol and xylene. For IHC staining, slices were subjected to antigen retrieval by heat treatment for 2.5 min after rehydration. Then, the slides were blocked in 20% goat serum for 30 min and incubated with primary antibodies overnight at 4°C in a dark room. Peroxidase bound to the antibody complex was labeled with a 3,3′-diaminobenzidine chromogen substrate solution provided with a Real^TM^ Envision^TM^ Detection System (DAKO, Copenhagen, Denmark).

Images were capture using kfbio scanner (KF-PRO-020) and immunoreactivity was evaluated by IHC profiler plugin in Image J. The staining was scored according to the percentage contribution of the positive staining area and intensity (high positive-4, positive-3, low positive-2, negative-1). The average staining score ( area × intensity) was generated from 4 different quadrants of each slide.

Data S1. (separate file)

Crotonylome data.
